# Prevalence of Contact Allergy to Isothiazolinones in Dermatitis Patients From 2000 to 2025: A Systematic Review and Meta‐Analysis

**DOI:** 10.1111/cod.70124

**Published:** 2026-03-10

**Authors:** Daniel Isufi, Kian Karimian, Mikkel Bak Jensen, Christoffer Kursawe Larsen, Rebekka Søgaard, Jeanne Duus Johansen, Jakob Ferløv Baselius Schwensen

**Affiliations:** ^1^ Department of Dermatology and Allergy Herlev and Gentofte—Copenhagen University Hospital Copenhagen Denmark; ^2^ National Allergy Research Centre, Department of Dermatology and Allergy Herlev and Gentofte Hospital Copenhagen Denmark; ^3^ Institute of Clinical Medicine, Faculty of Health and Medical Sciences University of Copenhagen Copenhagen Denmark

**Keywords:** allergic contact dermatitis, benzisothiazolinone, contact allergy, epidemiology, isothiazolinones, methylchloroisothiazolinone, methylisothiazolinone, prevalence

## Abstract

Isothiazolinones are employed in the preservation of cosmetic, consumer and industrial products, with the objective of preventing deterioration and spoilage. However, the utilization of isothiazolinones is associated with an elevated risk of developing contact allergy (CA). Herein, we assess the epidemiology of CA to isothiazolinones among dermatitis patients from year 2000 onwards. We systematically searched PubMed, Embase, and Web of Science from 1 January 2000 to 19 April 2025 yielding 115 studies comprising 1 514 781 dermatitis patients. The prevalence of CA to methylchloroisothiazolinone/methylisothiazolinone (MCI/MI) was 4.58%, methylisothiazolinone (MI) was 5.48%, and benzisothiazolinone (BIT) was 2.09%. The clinical relevance ranged from 60.1% for MCI/MI, 55.6% for MI, and 35.3% for BIT. Asia and North and South America exhibited the highest rates of CA to isothiazolinones, whereas Europe showed lower rates. These findings underscore the efficacy of proactive risk management for post‐marketed substances such as MI, underscoring substantial regional variations in usage patterns, which are contingent on the strictness or permissiveness of their incorporation into everyday consumer products. There is an indication of a decline, particularly regarding MI and MCI/MI. However, there has been an increase in the use of substances such as BIT, which necessitates enhanced surveillance measures.

**Abbreviations**
AXIS: appraisal tool for cross‐sectional studies
BIT: benzisothiazolinone
CA: contact allergy
CI: confidence interval
MCI/MI: methylchloroisothiazolinone/methylisothiazolinone
MI: methylisothiazolinone
n: number
PPT: positive patch test
PRISMA: preferred reporting items for systematic reviews and meta‐analyses
PROSPERO: International Prospective Register of Systematic Reviews

## 1 Introduction

Isothiazolinones have for decades been used to prevent deterioration and spoilage of cosmetic, consumer and industrial products [1]. However, as with all preservatives (biocides), there is an inherent risk of developing contact allergy (CA) to these substances when using them. Over the years, we have seen several trends of high prevalence of CA to preservatives, such as formaldehyde, methylchloroisothiazolinone (later combined with methylisothiazolinone, MI, in a 3:1 ratio), methyldibromoglutaronitrile, MI as a stand‐alone preservative and more recently benzisothiazolinone (BIT). The most recognised outbreak involved MI, where a rapid worldwide increase in cases of CA to MI from 2010 onward was observed, mainly caused by the use of MI as a preservative in cosmetic products including wet wipes. Several large studies using retrospective surveillance data from Europe and North America have highlighted and characterised this MI CA outbreak [2, 3, 4]. Further, we have witnessed the same pattern in prospectively collected data in for example cross‐sectional studies [3, 5]. Political, media, and industrial awareness of these historical outbreaks often leads to restrictions and more regulated use, which subsequently reduces exposure and results in fewer relevant cases in the general population. However, despite regional (EU) legislative restrictions on the use of MI in cosmetic products, the use of isothiazolinones—particularly BIT—in consumer and non‐cosmetic products may be on the rise and could pose an unforeseen challenge in the EU [6]. In other parts of the Western world where timely restrictions are not in place, we continue to observe unprecedentedly high levels of sensitization to MI [2].

Herein, we want to examine the trend and epidemiology of sensitization to isothiazolinones from year 2000 onward including any regional and time‐dependent differences, applying the same methodological approach as for previous meta‐analyses [7, 8, 9].

## 2 Methods

Before initiation, a study protocol was registered on the Prospective Register of Systematic Reviews (PROSPERO; CRD420251034451). The study adhered to the Preferred Reporting Items for Systematic Review and Meta‐Analysis (PRISMA) guidelines [10].

### 2.1 Literature Search

Three databases (PubMed, Embase, and Web of Science) were searched with inception through 1st January 2000 to 19th April 2025 using the search string available in Table S1. All titles and abstracts were extracted and compiled into the web‐based screening tool Rayyan [11], and duplicates were manually removed.

### 2.2 Inclusion and Exclusion Criteria

Two authors (D.I. and K.K.) independently assessed studies for eligibility. Inclusion criteria were: (I) Original studies, (II) written in any language, (III) including ≥ 100 consecutively patch tested dermatitis patients who were patch tested with isothiazolinones (MI, MCI/MI and BIT) of any concentration and vehicle.

Exclusion criteria were: (I) studies investigating site‐specific dermatitis, for example, hand dermatitis, (II) studies examining samples from the general population, (III) studies including < 100 patients, and (IV) conference abstract or letters to the editor. Octylisothiazolinone (OIT) was excluded, as OIT is less consistently included in baseline and extended patch test series and is primarily associated with specific industrial exposures. If duplicate publications involving the same population were retrieved, the most comprehensive report was selected for inclusion. When eligibility could not be determined from the title or abstract alone, the study was retained for full‐text assessment.

### 2.3 Data Extraction and Quality Assessment

Data were extracted by D.I. and K.K. to pre‐defined table with data on the author, publication year, study period, study country, concentration, vehicle, number of positive patch tests (PPTs), sex distribution (male, %), age [mean, standard deviation (SD)], patients with atopic dermatitis (AD, %), and clinically relevant PPTs (%). The study quality of cross‐sectional studies was assessed using the Appraisal tool for Cross‐Sectional Studies (AXIS) [12].

### 2.4 Statistical Analysis

Statistical analyses were performed using R in RStudio (version 2025.05.0 Build 496). Pooled prevalence estimates and associated 95% confidence intervals (CIs) were calculated using a random‐effects model with the inverse variance method for weighting. Between‐study heterogeneity was estimated using the DerSimonian–Laird estimator for tau^2^, and the Jackson method was used to compute CIs for both tau^2^ and tau. The *I* ^2^ statistic, which quantifies the proportion of total variation due to heterogeneity rather than chance, was calculated based on the Q statistic. All proportions were logit‐transformed prior to meta‐analysis to stabilise variance and minimise the influence of extreme proportions. For individual studies, 95% CIs were calculated using the Clopper–Pearson exact method.

In studies that reported multiple concentrations for the same vehicle, the data were extracted separately. Consequently, the same author and year may appear multiple times in the analyses. When separate extraction was not possible, the data were grouped and included in the overall analysis used to categorise each allergen but excluded from the sub‐analyses.

## 3 Results

### 3.1 Qualitative Assessment of the Included Studies

#### 3.1.1 Eligible Studies

In total, 4805 articles (PubMed = 1717, Embase = 2184, and Web of Science = 904) were identified. After removing duplicates, a total of 3213 non‐duplicate articles were screened based on title of abstract. Of these, 145 were included for full‐text assessment. Based on the full‐text article, 30 were excluded with reasons yielding 115 articles included in the meta‐analysis (Figure S1).

#### 3.1.2 Characteristics of the Included Studies

A total of 115 studies [3, 4, 5, 13, 14, 15, 16, 17, 18, 19, 20, 21, 22, 23, 24, 25, 26, 27, 28, 29, 30, 31, 32, 33, 34, 35, 36, 37, 38, 39, 40, 41, 42, 43, 44, 45, 46, 47, 48, 49, 50, 51, 52, 53, 54, 55, 56, 57, 58, 59, 60, 61, 62, 63, 64, 65, 66, 67, 68, 69, 70, 71, 72, 73, 74, 75, 76, 77, 78, 79, 80, 81, 82, 83, 84, 85, 86, 87, 88, 89, 90, 91, 92, 93, 94, 95, 96, 97, 98, 99, 100, 101, 102, 103, 104, 105, 106, 107, 108, 109, 110, 111, 112, 113, 114, 115, 116, 117, 118, 119, 120, 121, 122, 123, 124] were included, comprising 1 514 781 patients. Of these, 287 232 (19.0%) were males (*n* = 87 studies [3, 13, 17, 18, 20, 22, 23, 24, 25, 28, 29, 31, 32, 33, 34, 35, 36, 37, 38, 40, 41, 42, 43, 44, 45, 46, 47, 50, 52, 53, 54, 57, 60, 61, 62, 63, 65, 67, 68, 69, 70, 71, 72, 73, 74, 75, 76, 78, 79, 80, 81, 82, 83, 84, 85, 86, 88, 90, 91, 92, 93, 94, 95, 97, 98, 99, 100, 103, 104, 105, 106, 108, 109, 110, 112, 113, 114, 115, 116, 117, 118, 119, 120, 121, 122, 124]). The mean age across studies was 34.7 years (standard deviation: 15.2 years, *n* = 24 studies [13, 17, 33, 46, 47, 53, 57, 64, 65, 68, 72, 74, 83, 85, 90, 95, 99, 109, 112, 115, 116, 120, 124, 125]), while the median age was 41.0 years based on studies that reported median age with ranges (*n* = 27 studies [18, 31, 33, 36, 38, 44, 54, 62, 69, 71, 73, 76, 77, 84, 90, 93, 97, 98, 105, 106, 110, 112, 114, 115, 117, 120, 121]). Across 22 studies [21, 22, 23, 24, 25, 33, 34, 38, 40, 44, 52, 57, 67, 71, 75, 81, 88, 98, 99, 102, 109, 116], 56 806 (20.4%) of 277 504 patients reported a history of AD. The studies were conducted in Europe (*n* = 63 studies [3, 4, 5, 13, 14, 15, 16, 18, 19, 21, 22, 23, 24, 26, 27, 28, 29, 30, 32, 33, 34, 35, 36, 37, 39, 40, 41, 42, 45, 47, 48, 50, 51, 55, 56, 57, 60, 61, 67, 69, 71, 75, 77, 86, 88, 91, 92, 93, 96, 97, 98, 100, 101, 102, 103, 104, 107, 109, 113, 116, 119, 122, 125]), Middle East (*n* = 14 studies [38, 46, 66, 76, 87, 89, 90, 95, 105, 111, 112, 114, 120, 124]), North America (*n* = 11 studies [44, 52, 58, 70, 74, 81, 84, 85, 94, 121, 123]), Southeast Asia (*n* = 10 studies [53, 54, 64, 65, 68, 78, 83, 99, 106, 115]), Oceania (*n*AD (random effects): = 6 studies [20, 31, 59, 73, 108, 118]), South America (*n* = 4 studies [25, 72, 110, 117]), East Asia (*n* = 4 studies [17, 49, 63, 79]), Baltics (*n* = 1 study [43]), South Africa (*n* = 1 study [62]), and one study [82] across three continents (Table S2).

Based on the AXIS assessment, most studies were marked as acceptable risk of bias (Table S3).

### 3.2 Quantitative Assessment

#### 3.2.1 CA To Isothiazolinones in All Patients

**FIGURE 1:**
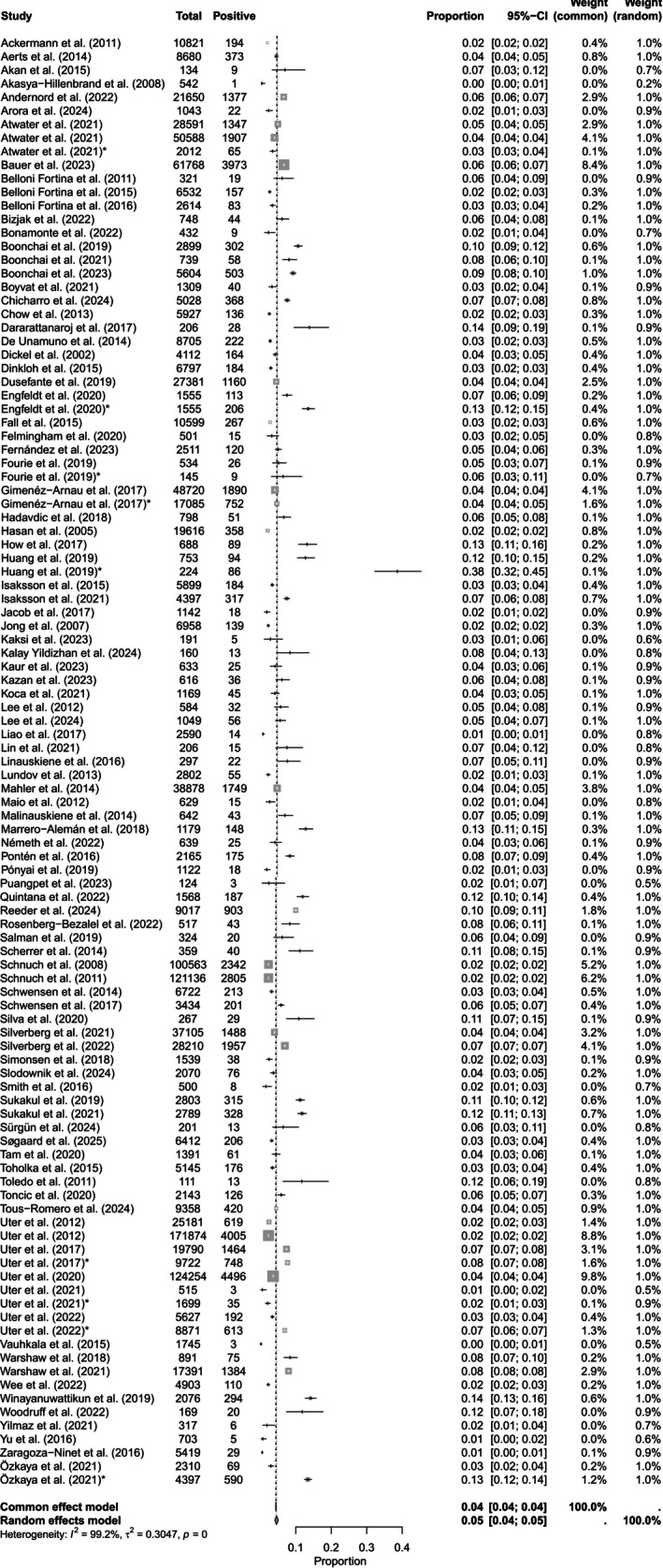
Forest plot of the prevalence of contact allergy to methylchloroisothiazolinone/methylisothiazolinone in dermatitis patients. Proportion meta‐analysis plot with random effects of dermatitis patients tested with methylchloroisothiazolinone/methylisothiazolinone. The figure displays the author, publication year, prevalence, confidence intervals, and the pooled prevalence for all studies.

**FIGURE 2:**
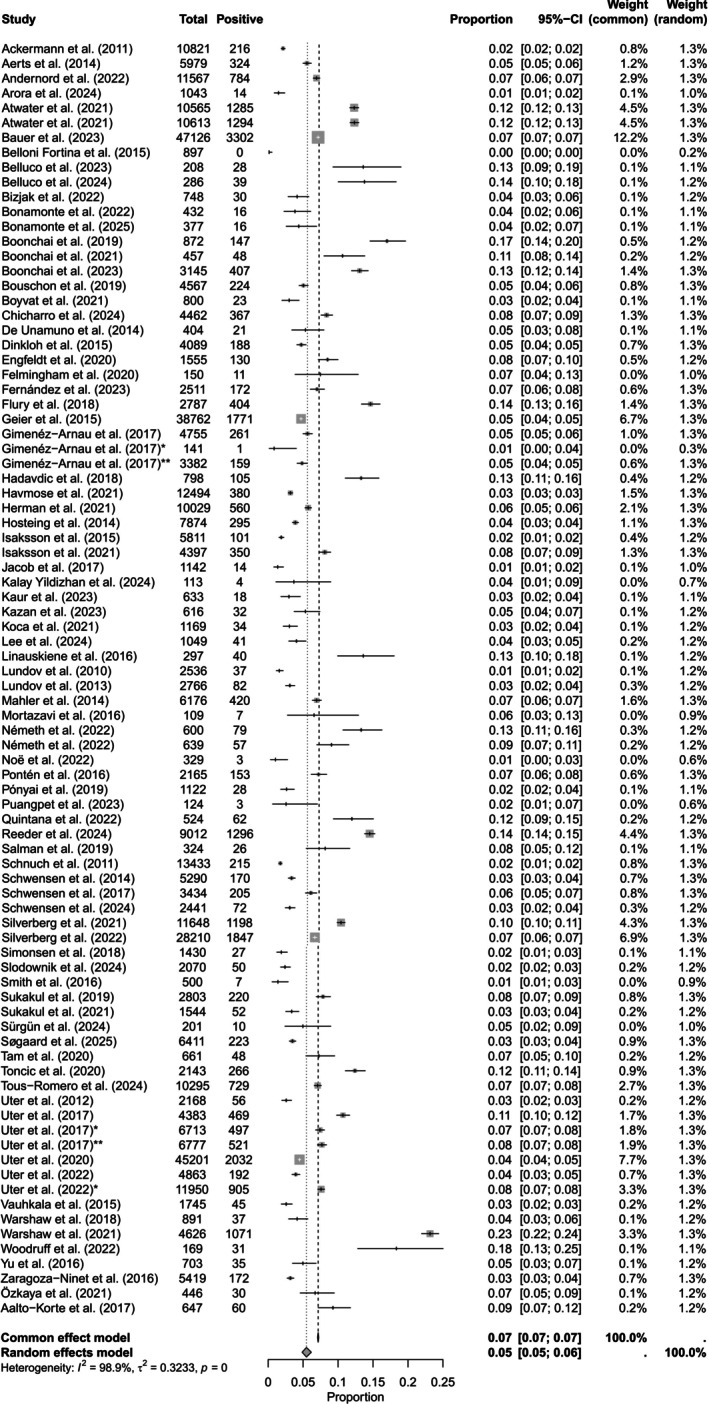
Forest plot of the prevalence of contact allergy to methylisothiazolinone in dermatitis patients. Proportion meta‐analysis plot with random effects of dermatitis patients tested with methylisothiazolinone. The figure displays the author, publication year, prevalence, confidence intervals, and the pooled prevalence for all studies.

**FIGURE 3:**
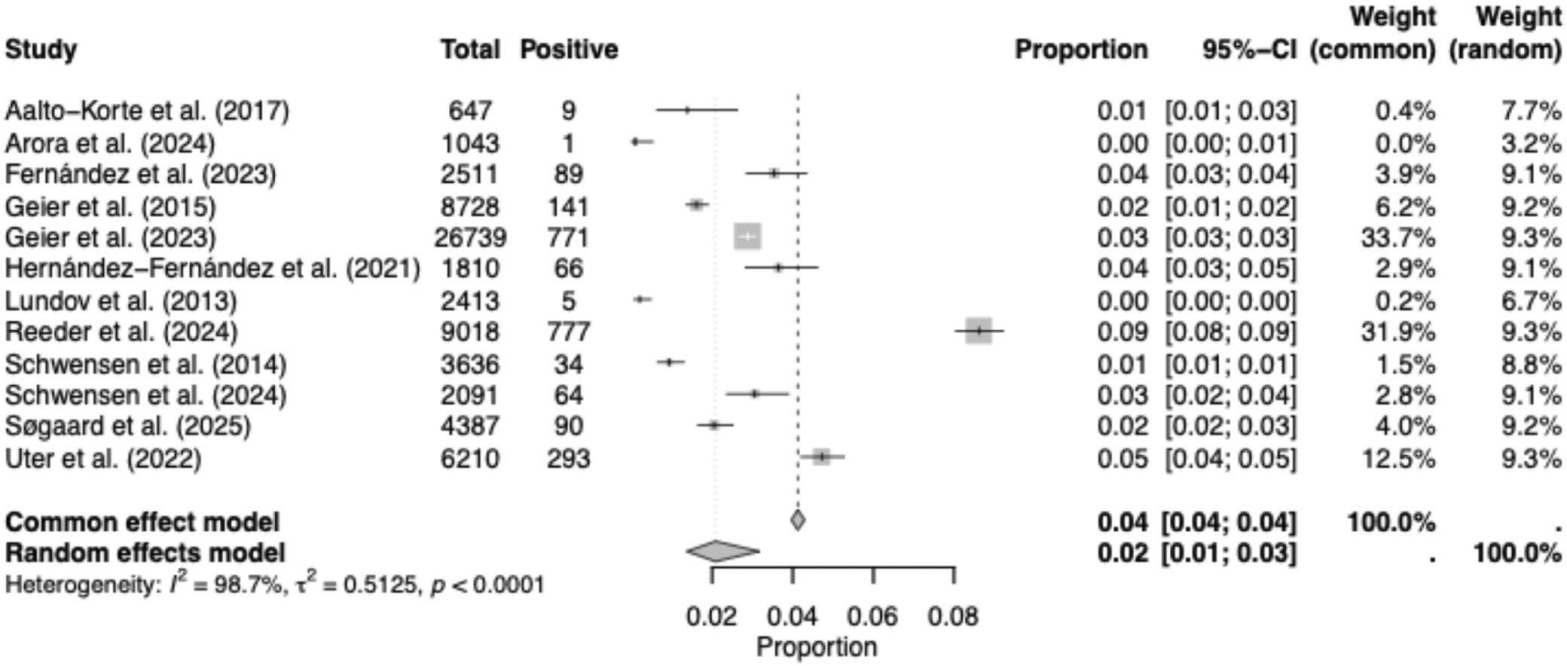
Forest plot of the prevalence of contact allergy to benzisothiazolinone in dermatitis patients. Proportion meta‐analysis plot with random effects of dermatitis patients tested with benzisothiazolinone. The figure displays the author, publication year, prevalence, confidence intervals, and the pooled prevalence for all studies.

The prevalence of CA to MCI/MI was reported in 1 196 076 patients (*n* = 97 studies [3, 4, 13, 14, 15, 16, 17, 18, 19, 20, 21, 22, 23, 24, 25, 26, 28, 29, 30, 31, 32, 33, 35, 36, 37, 38, 40, 41, 42, 43, 44, 45, 47, 48, 49, 50, 52, 53, 54, 55, 57, 58, 60, 61, 62, 63, 64, 65, 66, 67, 68, 69, 70, 71, 72, 73, 74, 75, 76, 78, 79, 80, 81, 82, 83, 84, 85, 86, 87, 89, 90, 92, 93, 94, 95, 96, 98, 99, 100, 102, 103, 105, 106, 108, 109, 111, 112, 113, 114, 115, 118, 119, 120, 121, 122, 123, 124]) with 54 780 PPTs yielding a pooled prevalence of 4.58% (95% CI, 4.12–5.08). For MI, 435564 patients (*n* = 80 studies [3, 4, 5, 14, 15, 19, 21, 23, 24, 26, 27, 28, 29, 30, 34, 37, 39, 40, 41, 42, 43, 44, 45, 46, 50, 51, 52, 55, 56, 57, 58, 59, 61, 65, 66, 68, 69, 70, 71, 73, 74, 75, 76, 77, 78, 81, 82, 83, 84, 85, 88, 89, 90, 92, 93, 94, 96, 97, 98, 100, 102, 103, 104, 106, 108, 109, 110, 111, 112, 115, 117, 118, 119, 120, 121, 122, 123, 124, 125]) were tested with 23 869 PPTs yielding a pooled prevalence of 5.48% (95% CI, 4.86–6.17). For BIT, 69233 patients (*n* = 13 studies [5, 21, 23, 39, 56, 84, 91, 101, 107, 109, 121, 123, 125]) were tested with 1447 PPTs with a pooled prevalence of 2.09% (95% CI, 1.37–3.17) (Table 1, Figures 1, 2, 3, Figures S2–S4).

**TABLE 1:** Proportion of positive patch tests (PPTs) to isothiazolinones and clinical relevance in all patients.

Allergen	Studies [*n*]	Patients undergoing patch testing [*n*]	PPTs [% (95% CI)]	p for Q‐test	*I* ^2^ [% (95% CI)]	Clinical relevance studies [*n*]	Clinical relevance [% (95% CI)]
MCI/MI	97	1 196 076	4.58 [4.12; 5.08]	0	99.2 [99.1; 99.2]	26	60.1 [50.4; 69.9]
MI	80	435 564	5.48 [4.86; 6.17]	0	98.9 [98.8; 99.0]	23	55.6 [45.5; 65.8]
BIT	13	69 233	2.09 [1.37; 3.17]	< 0.01	98.7 [98.4; 99.0]	4	35.3 [14.7; 55.8]

**Abbreviations:** BIT, benzisothiazolinone; CI, confidence interval; MCI/MI, methylchloroisothiazolinone/methylisothiazolinone; MI, methylisothiazolinone; *n*, number; PPTs, positive patch tests.

The clinical relevance was reported in 26 [4, 13, 17, 21, 23, 31, 44, 45, 47, 52, 57, 72, 73, 76, 79, 81, 85, 87, 94, 103, 106, 113, 118, 123, 124, 125], 23 [4, 21, 23, 27, 40, 45, 51, 52, 56, 57, 59, 73, 76, 81, 85, 94, 103, 106, 118, 123, 124, 125], and 4 [23, 91, 123, 125] studies for MCI/MI, MI, and BIT, respectively. The clinical relevance of PTTs was 60.1% (95% CI, 50.4–69.9) for MCI/MI, 55.6% (95% CI, 45.5–65.8) for MI, and 35.3% (95% CI, 14.7–55.8) for BIT (Table 1).

The rates of CA to MCI/MI 0.01% aqueous (aq.) were reported among 820 832 patients (*n* = 39 studies [3, 14, 15, 16, 18, 19, 20, 21, 23, 24, 26, 28, 29, 31, 33, 35, 36, 44, 47, 50, 53, 54, 55, 57, 58, 60, 61, 62, 63, 67, 70, 71, 74, 85, 86, 96, 99, 102, 125]), yielding a pooled prevalence rate of 3.95% (95% CI, 3.45–4.43). For MCI/MI 0.02% aq., the prevalence was 6.61% (95% CI, 5.73–7.54) based on 187 466 patients (*n* = 35 studies [17, 22, 32, 37, 41, 43, 50, 55, 62, 63, 64, 65, 66, 68, 76, 78, 81, 82, 83, 90, 92, 93, 94, 98, 106, 108, 109, 111, 112, 115, 118, 120, 121, 123, 124]). For MI 0.05% aq., the rate was 4.66% (95% CI, 3.61–5.37) based on 123 034 patients (*n* = 11 studies [3, 15, 19, 24, 26, 28, 29, 39, 46, 50, 96]), and for MI 0.2% aq., the rate of CA was 6.44% (95% CI, 5.47–7.49) based on 159 662 patients (*n* = 46 studies [4, 5, 21, 34, 41, 43, 45, 50, 55, 57, 58, 59, 61, 65, 66, 68, 69, 71, 74, 76, 78, 81, 82, 83, 85, 89, 92, 93, 94, 96, 98, 102, 104, 106, 108, 109, 110, 111, 112, 115, 118, 120, 121, 123, 124, 125]).

For BIT 0.05% petrolatum (pet.), the prevalence was 1.4%, while BIT 0.1% pet. revealed a prevalence of CA of 2.98% (95% CI, 1.74–4.54) based on 647 patients. A significant difference between the above‐mentioned concentrations was found for all three allergens (chi‐square test; *p* < 0.01) (Table 2).

**TABLE 2:** Proportion of positive patch tests (PPTs) to isothiazolinones in different concentrations.

Allergen, [conc.] vehicle	Studies [*n*]	Patients tested [*n*]	PPTs [% (95% CI)]	*p* for Cochrane Q‐test	*I* ^2^ [% (95% CI)]	Chi‐squared *p*
MCI/MI, 0.01 aq	39	820 832	3.95 [3.45; 4.43]	< 0.01	99.0 [99.0; 99.1]	< 0.01
MCI/MI, 0.02 aq	35	187 466	6.61 [5.73; 7.54]	< 0.01	98.2 [98.0; 98.3]	
MI, 0.2 aq	46	159 662	6.44 [5.47; 7.49]	< 0.01	98.4 [98.3; 98.5]	< 0.01
MI, 0.05 aq	11	123 034	4.66 [3.61; 5.37]	< 0.01	98.5 [98.2; 98.7]	
BIT, 0.1 pet	10	64 950	2.98 [1.74; 4.54]	< 0.01	98.9 [98.8; 99.1]	< 0.01
BIT, 0.05 pet	1	647	1.4 [NA]	NA	NA	

Abbreviations: aq, aqua; BIT, benzisothiazolinone; CI, confidence interval; MCI/MI, methylchloroisothiazolinone/methylisothiazolinone; MI, methylisothiazolinone; NA, not available; *n*, number; PPTs, positive patch tests.

#### 3.2.2 CA To Isothiazolinones in Patients With and Without AD

For MCI/MI, a total of 18 studies [21, 22, 23, 24, 25, 38, 40, 44, 52, 57, 67, 71, 75, 81, 98, 99, 102, 109] compared 19 174 patients with and 80 131 patients without AD and found pooled prevalence rates of 3.77% (95% CI, 3.00–4.61) for AD and 3.31% (95% CI, 2.69–3.98) for patients without AD (chi‐square test; *p* = 0.02) (Table 3).

**TABLE 3:** Comparison of positive patch test (PPTs) reactions to isothiazolinones in patients with and without atopic dermatitis (AD).

Allergen	Studies [*n*]	Non‐AD		AD		AD vs. non‐AD (random effects): Chi‐squared *p*
		Non‐AD patients [*n*]	PPTs [% (95% CI)]	AD patients [*n*]	PPTs [% (95% CI)]	
MCI/MI	18	80 131	3.31 [2.69; 3.98]	19 174	3.77 [3.00; 4.61]	0.02
MI	16	32 002	5.08 [3.15; 7.45]	9525	4.29 [2.87; 5.98]	< 0.05
BIT	2	4929	2.28 [0.30; 6.03]	1218	1.01 [0.35; 1.98]	< 0.05

Abbreviations: AD, atopic dermatitis; BIT, benzisothiazolinone; CI, confidence interval; MCI/MI, methylchloroisothiazolinone/methylisothiazolinone; MI, methylisothiazolinone; *n*, number; PPTs, positive patch tests.

For MI, 16 studies [21, 23, 24, 33, 34, 44, 52, 57, 71, 75, 81, 88, 98, 102, 109, 116] compared 9525 patients with and 32 002 patients without AD with prevalences of 4.29% (95% CI, 2.87–5.98) for AD and 5.08% (95% CI, 3.15–7.45) for patients without AD. A significant difference was found between patients with and without AD (chi‐square test; *p* < 0.05) (Table 3).

For BIT, two studies [23, 109] compared 1218 patients with and 4929 patients without AD with prevalences of 1.01% (95% CI, 0.35–1.98) for AD and 2.28% (95% CI, 0.30–6.03) for patients without AD. A significant difference was found between patients with and without AD (chi‐square test; *p* < 0.05) (Table 3).

#### 3.2.3 CA To Isothiazolinones According to Geographical Regions

Based on geographical regions, the highest rates of CA to MCI/MI were found in South America (11.02% [95% CI, 8.80–13.73], *n* = 2 studies [25, 72]), Southeast Asia (8.89% [95% CI, 8.53–9.27], *n* = 10 studies [53, 54, 64, 65, 68, 78, 83, 99, 106, 115]), East Asia (5.53% [95% CI, 4.89–6.25], *n* = 4 studies [17, 49, 63, 79]), and North America (5.48% [95% CI, 5.38–5.58], *n* = 11 studies [44, 52, 58, 70, 74, 81, 84, 85, 94, 121, 123]). The lowest rate was found in Oceania (3.08% [95% CI, 2.80–3.39], *n* = 5 studies [20, 31, 73, 108, 118]), and Europe (3.46% [95% CI, 3.42.3.49], *n* = 49 studies [3, 4, 13, 14, 15, 16, 18, 19, 21, 22, 23, 24, 26, 28, 29, 30, 32, 33, 35, 36, 37, 40, 41, 42, 45, 47, 48, 50, 55, 57, 60, 61, 67, 69, 71, 75, 86, 92, 93, 96, 98, 100, 102, 103, 109, 113, 119, 122, 125]) (Table 4).

**TABLE 4:** Proportion of positive patch tests (PPTs) to isothiazolinones by geographical region.

Europe		Middle East		North America		East Asia		Southeast Asia		Oceania		South Africa		South America	
Studies [*n*]	PPTs [% (95% CI)]	Studies [*n*]	PPTs [% (95% CI)]	Studies [*n*]	PPTs [% (95% CI)]	Studies [*n*]	PPTs [% (95% CI)]	Studies [*n*]	PPTs [% (95% CI)]	Studies [*n*]	PPTs [% (95% CI)]	Studies [*n*]	PPTs [% (95% CI)]	Studies [*n*]	PPTs [% (95% CI)]
MCI/MI															
49	3.46 [3.42; 3.49]	13	3.81 [3.45; 4.21]	11	5.48 [5.38; 5.58]	4	5.53 [4.89; 6.25]	10	8.89 [8.53; 9.27]	5	3.08 [2.80; 3.39]	3	4.43 [4.14; 4.74]	2	11.02 [8.80; 13.73]
MI															
46	5.20 [5.12; 5.27]	9	3.69 [3.24; 4.21]	11	10.30 [10.10; 10.52]	NA	NA	6	9.80 [9.21; 10.44]	4	10.26 [9.42; 11.17]	NA	NA	2	13.56 [10.82; 16.87]
BIT															
10	2.64 [2.51; 2.77]	NA	NA	3	7.73 [7.23; 8.27]	NA	NA	NA	NA	NA	NA	NA	NA	NA	NA

Abbreviations: BIT, benzisothiazolinone; CI, confidence interval; MCI/MI, methylchloroisothiazolinone/methylisothiazolinone; MI, methylisothiazolinone; NA, not available; n, number; PPTs, positive patch tests.

For MI, the highest prevalence of CA was found in South America (13.56% [95% CI, 10.82–16.87], *n* = 2 studies [110, 117]), North America (10.30% [95% CI, 10.10–10.52], *n* = 11 studies [44, 52, 58, 70, 74, 81, 84, 85, 94, 121, 123]), Oceania (10.26% [95% CI, 9.42–11.17], *n* = 4 studies [59, 73, 108, 118]), and Southeast Asia (9.80% [95% CI, 9.21–10.44], *n* = 6 studies [65, 68, 78, 83, 106, 115]). The lowest rates were found in Europe (5.20% [95% CI, 5.12–5.27], *n* = 46 studies [3, 4, 5, 14, 15, 19, 21, 23, 24, 26, 27, 28, 29, 30, 34, 37, 39, 40, 41, 42, 45, 50, 51, 55, 56, 57, 61, 69, 71, 75, 77, 88, 92, 93, 96, 97, 98, 100, 102, 103, 104, 109, 116, 119, 122, 125]), and the Middle East (3.69% [3.24–4.21], *n* = 9 studies [46, 66, 76, 89, 90, 111, 112, 120, 124]) (Table 4).

The prevalence of CA to BIT according to geographical regions could only be assessed in North America (7.73% [95% CI, 7.23–8.27], *n* = 3 studies [84, 121, 123]), and Europe (2.64% [95% CI, 2.51–2.77], *n* = 10 studies [5, 21, 23, 39, 56, 91, 101, 107, 109, 125]) (Table 4).

#### 3.2.4 CA To Isothiazolinones by Study Period

For MCI/MI, 71 studies [3, 4, 13, 14, 15, 16, 17, 18, 19, 20, 21, 22, 23, 24, 25, 26, 28, 29, 30, 31, 33, 35, 36, 37, 38, 40, 41, 42, 43, 44, 45, 47, 49, 50, 52, 53, 54, 55, 57, 58, 60, 61, 62, 63, 64, 65, 66, 67, 68, 69, 70, 71, 72, 73, 74, 75, 76, 81, 82, 83, 85, 86, 87, 92, 94, 95, 98, 99, 103, 113, 114] had a study period before year 2018 with a pooled prevalence of 4.17% (95% CI, 3.63–4.79), and 6 studies [93, 96, 109, 111, 121, 124] after 2018 with a prevalence of 4.44% (95% CI, 3.27–5.99).

For MI, 50 studies [3, 4, 14, 15, 19, 21, 23, 24, 26, 27, 28, 29, 30, 34, 37, 39, 40, 41, 42, 43, 44, 45, 46, 50, 51, 52, 55, 56, 57, 58, 59, 61, 65, 66, 68, 69, 70, 71, 73, 74, 75, 76, 81, 82, 83, 85, 92, 94, 98, 103] reported data before year 2018 with a pooled prevalence of 5.10% (95% CI, 4.20–6.18), and 12 studies [5, 93, 96, 109, 110, 111, 112, 117, 119, 121, 122, 124] after 2018 with a prevalence of 5.00% (95% CI, 3.61–6.88).

For BIT, 4 studies [21, 23, 39, 56] reported data before year 2018 with a pooled prevalence of 0.91% (95% CI, 0.50–1.66) and 3 studies [5, 109, 121] after 2018 with a prevalence of 3.75% (95% CI, 1.54–8.86).

The differences between the two time periods were significant for MCI/MI (chi‐squared test; *p* = 0.0117) and BIT (chi‐squared test; *p* = 0.0003), but not for MI (chi‐squared test; *p* = 0.2225) (Table 5).

**TABLE 5:** Proportion of positive patch tests (PPTs) according to study period before and after year 2018.

Allergen	Study period [year]	Studies [*n*]	PPTs [% (95% CI)]	Tau‐squared	Test for subgroup differences (random effects): Chi‐squared *p*
MCI/MI	Before 2018	71	4.17 [3.63; 4.79]	0.3479	0.0117
	After 2018	6	4.44 [3.27; 5.99]	0.1993	
	Overlapping^a^	32	5.77 [4.89; 6.80]	0.1647	
MI	Before 2018	50	5.10 [4.20; 6.18]	0.4720	0.2225
	After 2018	12	5.00 [3.61; 6.88]	0.2062	
	Overlapping^a^	23	6.27 [5.27; 7.44]	0.2769	
BIT	Before 2018	4	0.91 [0.50; 1.66]	0.3136	0.0003
	After 2018	3	3.75 [1.54; 8.86]	0.6486	
	Overlapping^a^	6	3.46 [2.59; 4.60]	0.0828	

**Abbreviations:** BIT, benzisothiazolinone; CI, confidence interval; MCI/MI, methylchloroisothiazolinone/methylisothiazolinone; MI, methylisothiazolinone; n, number; PPTs, positive patch tests.

^a^ Studies with study period overlapping 2018 where data could not be extracted separately were reported separately.

## 4 Discussion

This systematic review and meta‐analysis of 115 studies, encompassing 1 514 781 patients, consolidates and characterises the outbreak of CA to MI observed over the last decade and a half. The pooled prevalence of CA to MI and MCI/MI was high from a historical perspective, at 5.48% (95% CI, 4.86–6.17) and 4.58% (95% CI, 4.12–5.08), respectively. Thus, highlighting the problematic use of MI in water‐based products such as cosmetic products, consumer goods, and industrial products like water‐based paints [5, 126, 127]. All these product categories are ones with which the consumer may be in daily contact and therefore pose an ongoing risk of developing CA and clinically relevant allergic contact dermatitis. This leads to an alarming clinical relevance of around 60% for both MI and MCI/MI showing the current and global problem with sensitization to MI and MCI/MI. In contrast, the ban and legislative restrictions on the use of MI in leave‐on and rinse‐off cosmetic products in the EU have already led to a decrease in the prevalence of sensitization to MI and MCI/MI, as well as a reduction in clinical relevance, dropping from 72.7% in 2015 to 41.7% in 2022 according to large cross‐sectional studies [4, 5]. Such post‐marketing restrictions to limit the use of MI to 15 ppm in rinse‐off water‐based cosmetic products were initiated and finalised by the European Commission, with full implementation by 2018 approximately 3 years after the peak of the MI sensitization outbreak. This underscores that the basis for the strict ban and regulatory measures was established years earlier, notably through contributions from members of the ESCD and the 2013 SCCS opinion [128, 129], which may have encouraged some degree of self‐regulation by the cosmetic industry, particularly in restricting the use of MI in wet wipes and leave‐on cosmetic products [3]. Moreover, while we found differences in isothiazolinones sensitization between patients with and without AD, these findings should be interpreted cautiously, as disease severity and treatment was not consistently reported. Thus, findings reflect associations rather than causal relationships. Our data further highlight the importance of timely post‐market risk management, as regional differences in the prevalence of sensitization to isothiazolinones are observed, consistent with findings from a previous American‐German collaboration [2]. Herein, we report a European prevalence of 3.46% (95% CI, 3.42–3.49) for MCI/MI and 5.20% (95% CI, 5.12–5.27) for MI, compared to 5.48% (95% CI, 5.38–5.58) and 10.30% (95% CI, 10.10–10.52), respectively, in North America, with even higher prevalences observed in other regions globally. Additionally, in a recent meta‐analysis investigating sensitization to MI, among other substances, in children, we observed the same pattern of regional differences, with a lower pooled prevalence of MI in Europe compared to North America [7]. The increasing use of BIT has also been mirrored in recent reports indicating rising rates of CA to BIT within the European Union, likely associated with its increased use in non‐cosmetic products [96, 107, 125, 130, 131]. This is in accordance with our data, which show a significant increase in the pooled prevalence of sensitization to BIT from 0.91% (95% CI, 0.50–1.66) before 2018 to 3.75% (95% CI, 1.54–8.86) after 2018. However, considering the pooled prevalence of BIT in different regions, we observe the same pattern as for MI and MCI/MI, with a higher pooled prevalence in North America compared to Europe. Two explanations may account for this: (I) BIT is permitted in cosmetic products in the United States and Canada, whereas (II) BIT is not allowed for use in cosmetic products in the European Union following advice from the SCCS^129^. Despite regional differences in pooled prevalences of BIT, we emphasise the need for awareness and continuous regional monitoring of sensitization rates to BIT, as well as thorough investigation of its clinical relevance. Based on our presented data, we currently find that only 35.3% (95% CI, 14.7–55.8) of cases are relevant to the current disease, which may indicate overlooked relevance or insufficient labeling of consumer and industrial products. Further, BIT is known to be a problematic allergen for patch testing, often eliciting questionable, doubtful or irritant‐type reactions, making interpretation challenging [107].

The strengths of this study are (I) the extensive literature search including 115 studies of 1 514 781 patients, (II) sub‐analysis based on geographical region, study period, AD, and concentration, and (III) meta‐analysis on clinically relevant PPTs. However, some limitations should be noted: (I) only a few studies reported clinical relevance, (II) few studies reported data on patients with and without AD, (III) rates of CA to isothiazolinones relied on a few studies in some geographical regions, and (IV) systematic comparison between occupational and non‐occupational cases of CA was limited by inconsistent reporting and variability in exposure assessment methods and meta‐analysis could not be conducted.

## 5 Conclusion

This meta‐analysis highlights the effectiveness of proactive risk management for post‐marketed substances such as MI, demonstrating substantial regional differences based on the strictness or permissiveness of their use in everyday consumer products, such as cosmetic products. Meta‐analysis revealed rates of CA to MCI/MI of 4.58%, MI of 5.48%, and BIT of 2.09%. In the EU, lower prevalences of all isothiazolinones (MCI/MI: 3.46%, MI: 5.20%, BIT: 2.65%) have been observed compared to North America (MCI/MI: 5.48%, MI: 3.69%). Several of the manuscripts included herein show that surveillance data across Europe indicate a declining trend, particularly for MI and MCI/MI. However, substances such as BIT are on the rise, driven in Europe by its use in consumer products, while exposure patterns may be more complex in other parts of the world.

## Author Contributions

**Daniel Isufi:** conceptualization, investigation, writing – original draft, writing – review and editing, validation, methodology, project administration, data curation, supervision. **Mikkel Bak Jensen:** software, formal analysis, visualization, methodology, investigation, supervision. **Rebekka Søgaard:** conceptualization, investigation, writing – review and editing, data curation, methodology. **Kian Karimian:** investigation, writing – review and editing, methodology, data curation. **Jeanne Duus Johansen:** conceptualization, investigation, visualization, writing – review and editing, project administration, resources, supervision.

## Funding

This work was supported by The Danish Environmental Protection Agency under the Ministry of Environment of Denmark.

## Conflicts of Interest

Mr. Isufi, Mr. Karimian, Dr. Jensen, Dr. Larsen, Dr. Søgaard, and Dr. Johansen have no conflicts of interest to declare. Dr. Schwensen has previously served as speaker and investigator for Galderma and Sanofi‐Aventis.

## Supporting information


**Table S1:** Search string for databases.
**Table S2:** General characteristics of the included studies.
**Table S3:** Appraisal tool for cross‐sectional studies (AXIS) assessment of included studies.
**Figure S1:** The preferred reporting items for systematic reviews and meta‐analyses (PRISMA) flowchart.
**Figure S2:** Funnel plot of contact allergy to Methylchloroisothiazolinone/methylisothiazolinone in all patients.
**Figure S3:** Funnel plot of contact allergy to methylisothiazolinone in all patients.
**Figure S4:** Funnel plot of contact allergy to benzisothiazolinone in all patients.

**Data S1:** Supporting Information.

**Data S2:** Supporting Information.

## Data Availability

The data that support the findings of this study are available from the corresponding author upon reasonable request.
