# Supplementary material for: Prevalence of Contact Allergy to Isothiazolinones in Dermatitis Patients From 2000 to 2025: A Systematic Review and Meta‐Analysis
Source: Contact Dermatitis. 2026 Mar 10;94(6):577–91. doi: 10.1111/cod.70124 (PMC13139707; doi:10.1111/cod.70124)
Supplement: Supplementary file 1 — Table S1: Search string for databases. Table S2: General characteristics of the included studies. Table S3: Appraisal tool for cross‐sectional studies (AXIS) assessment of included studies. Figure S1: The preferred reporting items for systematic reviews and meta‐analyses (PRISMA) flowchart. Figure S2: Funnel plot of contact allergy to Methylchloroisothiazolinone/methylisothiazolinone in all patients. Figure S3: Funnel plot of contact allergy to methylisothiazolinone in all patients. Figure S4: Funnel plot of contact allergy to benzisothiazolinone in all patients. [file COD-94-577-s003.docx]

**SUPPLEMENTARY MATERIAL**

Prevalence of Contact Allergy to Isothiazolinones in Dermatitis Patients from 2000 to 2025: A Systematic Review and Meta-Analysis

*Daniel Isufi^1^, Kian Karimian^2^, Mikkel Bak Jensen^2,3^, Christoffer Kursawe Larsen^2^, Rebekka Søgaard^2,3^, Jeanne Duus Johansen^2,3^, Jakob Ferløv Baselius Schwensen^2,3^*

^1^ Department of Dermatology and Allergy, Herlev and Gentofte - Copenhagen University Hospital, Denmark
^2^ National Allergy Research Centre, Department of Dermatology and Allergy, Herlev and Gentofte Hospital, Denmark
^3^ Institute of Clinical Medicine, Faculty of Health and Medical Sciences , University of Copenhagen, Copenhagen, Denmark.
 **Corresponding author:**Daniel Isufi, Department of Dermatology and Allergy, Herlev and Gentofte - Copenhagen University Hospital, Gentofte Hospitalsvej 20A, Hellerup, Denmark
E-mail: [daniel.isufi@regionh.dk](mailto:daniel.isufi@regionh.dk)

| **Supplementary Table 1:** Search string for databases |
| --- |
| *("methylisothiazolinone" OR "2-Methyl-4-isothiazolin-3-one" OR "MI" OR "MIT" OR "2-Methyl-3(2H)-isothiazolone" OR "CAS 2682-20-4")*  *OR*  *("methylchloroisothiazolinone" OR "MCI" OR "MCI/MI" OR "Kathon CG" OR "Methylchloroisothiazolinone/Methylisothiazolinone" OR "5-Chloro-2-methyl-4-isothiazolin-3-one")*  *OR*  *("benzisothiazolinone" OR "BIT" OR "1,2-Benzisothiazolin-3-one" OR "Proxel”)*  *AND (“allergic contact dermatitis” OR “contact allergy” OR “contact dermatitis” OR “allergic reaction” OR “hypersensitivity” OR “contact sensitization” OR “contact sensitivity”)* |

| **Supplementary Table 2:** General characteristics of the included studies | | | | | | | | |
| --- | --- | --- | --- | --- | --- | --- | --- | --- |
| **Study name (year)** | **Study period (year)** | **Study country** | **Number of patients** | **Sex distribution (male, no.)** | **Sex distribution (Male, %)** | **Age (mean±SD)** | **History of atopic dermatitis (no.)** | **History of atopic dermatitis (%)** |
| Bonamonte (2025)[1] | 2016-2022 | Italy | 377 | 127 | 33.7 | 40 | 377 | 100 |
| Belluco (2024)[2] | 2020-2022 | Brazil | 286 | 59 | 20.63 | NA | 158 | 55.2 |
| Schwensen (2024)[3] | 2022-2022 | 8 European countries | 2554 | NA | NA | NA | NA | NA |
| Lee (2024)[4] | 2008-2021 | New Zealand | 1044 | 326 | 31.2 | NA | NA | NA |
| Tous-Romero (2024)[5] | 2018-2022 | Spain | 11327 | 3450 | 30.46 | NA | 1984 | 17.52 |
| Kalay Yildizhan (2024)[6] | 2013-2023 | Turkey | 160 | 69 | 43.1 | 12.4± 4.1 | 36 | 22.5 |
| Arora (2024)[7] | 2020-2023 | United States | 1043 | 271 | 26 | NA | 247 | 23.7 |
| Chicharro (2024)[8] | 2018-2020 | Spain | 5055 | 1519 | 30 | NA | 1168 | 23 |
| Reeder (2024)[9] | 2017-2020 | United States | 9028 | NA | NA | NA | NA | NA |
| Slodownik (2024)[10] | 2019-2022 | Israel | 2086 | 685 | 33 | 44.4 ± 20.01 | 396 | 19 |
| Boonchai (2023)[11] | 2001-2021 | Thailand | 5998 | 1279 | 21.3 | 41.9 ± 15.3 | 791 | 72.5 |
| Akasya-Hillenbrand (2008)[12] | 1996-1999 | Turkey | 542 | 239 | 44.1 | NA | 15 | 2.8 |
| Dickel (2002)[13] | 1990-1999 | Germany | 4112 | 1694 | 41.2 | NA | 789 | 19.2 |
| Sürgün (2024)[14] | 2018-2020 | Turkey | 201 | 84 | 41.8 | 41.1 ± 18.3 | NA | NA |
| Kazan (2023)[15] | 2020-2021 | Turkey | 616 | NA | NA | NA | NA | NA |
| Belluco (2023)[16] | 2020-2020 | Brazil | 208 | 39 | 18.75 | NA | 24 | 11.7 |
| Fernández (2023)[17] | 2019-2021 | Spain | 2511 | 761 | 30.3 | 48.5 ± 18.4 | 417 | 16.6 |
| Kaur (2023)[18] | 2017-2022 | Australia | 657 | 263 | 40 | NA | 228 | 34.7 |
| Geier (2023)[19] | 2002-2021 | 3 European countries | 212603 | NA | NA | NA | NA | NA |
| Puangpet (2023)[20] | NA | Thailand | 124 | 63 | 50.81 | NA | 50 | 40.3 |
| Kakşi (2023)[21] | 2015-2019 | Turkey | 191 | 77 | 40.3 | NA | NA | NA |
| Németh (2022)[22] | 2015-2019 | Hungary | 600 | 128 | 21.33 | NA | 11 | 1.8 |
| Quintana (2022)[23] | 2005-2018 | Spain | 1568 | 445 | 28.4 | NA | 222 | 14.2 |
| Németh (2022)[24] | 2007-2021 | Hungary | 639 | NA | NA | NA | 639 | 100 |
| Uter (2022)[25] | 2019-2020 | 12 European countries | 21633 | NA | NA | NA | NA | NA |
| Bauer (2023)[26] | 2011-2020 | 11 European countries | 89777 | 20735 | 23.1 | NA | 18515 | 20.62 |
| Wee (2022)[27] | 2007-2017 | Singapore | 4903 | 2043 | 41.7 | 40.07 ± 16.28 | 2529 | 51.6 |
| Bonamonte (2022)[28] | 2017-2018 | Italy | 432 | 200 | 46.3 | NA | 103 | 23.8 |
| Noë (2022)[29] | 2010-2019 | Belgium | 329 | 136 | 41 | NA | 179 | 54.41 |
| Uter (2022)[30] | 2019-2020 | 13 European countries | 22581 | NA | NA | NA | NA | NA |
| Rosenberg-Bezalel (2022)[31] | 2016-2018 | Israel | 517 | 133 | 25.73 | 42.9 ± 19 | 69 | 13.35 |
| Silverberg (2022)[32] | 2001-2018 | Canada and Unites States | 43572 | 14066 | 32.28 | NA | NA | NA |
| Bizjak (2022)[33] | 2019-2021 | Slovenia | 748 | 198 | 26.5 | NA | NA | NA |
| Andernord (2022)[34] | 2010-2017 | Sweden | 21663 | 6715 | 31 | NA | NA | NA |
| Uter (2021)[35] | 2007-2012 | Germany | 2213 | 797 | 36 | NA | 369 | 16.7 |
| Hernández-Fernández (2021)[36] | 2019-2020 | Spain | 4654 | 1396 | NA | NA | NA | NA |
| Atwater (2021)[37] | 1994-2016 | Canada and Unites States | 50799 | 17119 | 33.7 | 48 ± 16.9 | 11054 | 21.8 |
| Boyvat (2021)[38] | 2013-2019 | Turkey | 1309 | 545 | 41.6 | 40.9 ± 16.8 | 66 | 5.04 |
| Özkaya (2021)[39] | 1996-2019 | Turkey | 2310 | NA | NA | NA | NA | NA |
| Havmose (2021)[40] | 2005-2019 | Denmark | 12494 | 4032 | 32.27 | NA | 2585 | 20.69 |
| Yilmaz (2021)[41] | 1996-2017 | Turkey | 317 | NA | NA | NA | NA | NA |
| Woodruff (2022)[42] | 2013-2019 | United States | 169 | 62 | 36.7 | NA | 19 | 11.2 |
| Sukakul (2021)[43] | 2009-2018 | Thailand | 2789 | 667 | 23.9 | 42.7 ± 15.7 | NA | NA |
| Isaksson (2021)[44] | 2017-2017 | 10 countries | 4397 | 1264 | 28.75 | NA | NA | NA |
| Silverberg (2021)[45] | 2001-2016 | Canada and Unites States | 38482 | 12621 | 32.8 | NA | 5782 | 15.03 |
| Atwater (2021)[46] | 2005-2016 | Canada and Unites States | 28640 | 9247 | 32.29 | NA | NA | NA |
| Lin (2021)[47] | 1978-2018 | Taiwan | 4005 | 1209 | 30.19 | NA | NA | NA |
| Boonchai (2021)[48] | 2010-2019 | Thailand | 792 | 261 | 32.95 | NA | 88 | 11.11 |
| Herman (2021)[49] | 2014-2019 | Belgium | 10029 | NA | NA | NA | NA | NA |
| Koca (2021)[50] | 2015-2017 | Turkey | 1169 | 487 | 41.7 | NA | 48 | 4.1 |
| Toncic (2020)[51] | 2015-2017 | Croatia | 2143 | 563 | 26.27 | NA | 226 | 10.55 |
| Felmingham (2020)[52] | 1993-2017 | Australia | 511 | 199 | 38.9 | NA | 212 | 41.5 |
| Uter (2020)[53] | 2007-2018 | 3 European countries | 125436 | 46041 | 36.7 | NA | 28377 | 22.6 |
| Tam (2020)[54] | 2007-2016 | United States | 2373 | 632 | 26.6 | 47.7 ± 16.9 | 695 | 29.3 |
| Silva (2020)[55] | 2015-2017 | Brazil | 267 | 76 | 28.46 | 43 ± 16 | NA | NA |
| Pónyai (2019)[56] | 2015-2016 | Hungary | 1122 | 262 | 23.35 | NA | NA | NA |
| Warshaw (2021)[57] | 1996-2016 | United States | 48472 | 16233 | 33.49 | NA | NA | NA |
| Engfeldt (2020)[58] | 2014-2014 | Sweden | 1555 | 486 | 31.3 | NA | NA | NA |
| Sukakul (2019)[59] | 2006-2018 | Thailand | 2803 | 668 | 23.8 | 42.4 ± 15.5 | NA | NA |
| Dusefante (2019)[60] | 1996-2016 | Italy | 27381 | 8850 | 32.32 | NA | 1539 | 5.62 |
| Salman (2019)[61] | 2016-2018 | Turkey | 324 | NA | NA | NA | NA | NA |
| Boonchai (2019)[62] | 2005-2016 | Thailand | 3201 | 670 | 20.93 | 43.2 ± 15.7 | 214 | 6.69 |
| Winayanuwattikun (2019)[63] | 2007-2016 | Thailand | 2178 | NA | NA | 41.9 ± 15.6 | NA | NA |
| Huang (2019)[64] | 1996-2015 | Taiwan | 757 | 213 | 28.1 | NA | NA | NA |
| Fourie (2019)[65] | 2006-2018 | South Africa | 590 | 295 | 50 | NA | NA | NA |
| Hadžavdić (2018)[66] | 2015-2016 | Croatia | 798 | 198 | 24.81 | NA | 11 | 1.38 |
| Marrero-Alemán (2018)[67] | 2005-2015 | Spain | 1179 | 345 | 29.3 | NA | NA | NA |
| Flury (2018)[68] | 2011-2017 | Australia | 2787 | NA | NA | NA | NA | NA |
| Warshaw (2018)[69] | 2000-2014 | Canada and Unites States | 1069 | NA | NA | NA | NA | NA |
| Simonsen (2018)[70] | 2012-2016 | Denmark | 1573 | 568 | 36.1 | 12.5 ± 4.0 | 823 | 52.3 |
| Aalto-Korte (2017)[71] | 2012-2017 | Finland | 647 | NA | NA | NA | NA | NA |
| Uter (2017)[72] | 2013-2014 | 12 European countries | 31689 | NA | NA | NA | NA | NA |
| Schwensen (2017)[73] | 2015-2015 | 8 European countries | 3434 | NA | NA | NA | NA | NA |
| How (2017)[74] | 2011-2015 | Malaysia | 689 | 226 | 32.8 | NA | NA | NA |
| Dararattanaroj (2017)[75] | 2012-2015 | Thailand | 206 | 60 | 29.1 | 43.2 ± 15 | NA | NA |
| Jacob (2017)[76] | 2015-2015 | United States | 1142 | 387 | 33.89 | NA | 552 | 48.34 |
| Bouschon (2019)[77] | 2010-2015 | France | 4567 | NA | NA | NA | NA | NA |
| Gimenéz-Arnau (2017)[78] | 2009-2012 | 12 European countries | 59728 | 20068 | 33.6 | NA | 13439 | 22.5 |
| Liao (2017)[79] | 1987-2015 | Taiwan | 2590 | NA | NA | NA | NA | NA |
| Hasan (2005)[80] | 1995– 1997 and 2000–2002 | Finland | 19616 | NA | NA | NA | NA | NA |
| Fortina (2016)[81] | 2002-2013 | Italy | 2614 | 1188 | 45.45 | 5.3 ± 2.5 | 1283 | 49.1 |
| Mortazavi (2016)[82] | 2007-2009 | Iran | 109 | 37 | 33.9 | 14.4 ± 3.4 | NA | NA |
| Smith (2016)[83] | 2005-2014 | United Kingdom | 500 | 203 | 40.6 | NA | NA | NA |
| Yu (2016)[84] | 2005-2014 | Canada and Unites States | 703 | 208 | 29.59 | NA | NA | NA |
| Linauskienė (2016)[85] | 2014-2015 | Lithuania | 297 | 40 | 13.47 | NA | NA | NA |
| Zaragoza-Ninet (2016)[86] | 1996-2013 | Spain | 5419 | 2007 | 37 | NA | 1143 | 21.1 |
| Pontén (2016)[87] | 2012-2014 | Sweden | 2165 | 736 | 34 | NA | NA | NA |
| Fortina (2015)[88] | 2002-2010 | 11 European countries | 6708 | 2743 | 40.89 | NA | 2644 | 39.42 |
| Geier (2015)[89] | 2009-2013 | 3 European countries | 64039 | NA | NA | NA | NA | NA |
| Akan (2015)[90] | 2011-2012 | Turkey | 134 | 79 | 58.1 | NA | 134 | 100 |
| Isaksson (2015)[91] | 2003-2012 | Sweden | 5899 | 2222 | 37.67 | NA | NA | NA |
| Jong (2007)[92] | 2004-2005 | UK | 6958 | 2319 | 33.33 | NA | 362 | 5.2 |
| Schnuch (2008)[93] | 1996-2007 | 3 European countries | 100563 | 38060 | 37.8 | NA | NA | NA |
| Lundov (2010)[94] | 2006-2010 | Denmark | 2536 | 827 | 32.6 | NA | 432 | 17 |
| Fortina (2011)[95] | 2002-2008 | Italy | 321 | 144 | 44.9 | 2.25+0.46 | 137 | 42.7 |
| Fall (2015)[96] | 1991-1993. 1999-2001 and 2008-2010 | Sweden | 10599 | 3818 | 36.02 | NA | NA | NA |
| Toholka (2015)[97] | 2001-2010 | Australia | 5521 | 1865 | 35 | NA | 1576 | 30 |
| Vauhkala (2015)[98] | 2002-2013 | Finland | 1745 | NA | NA | NA | NA | NA |
| Dinkloh (2015)[99] | 2006-2011 | 3 European countries | 10124 | 3063 | 30.3 | NA | 1539 | 15.2 |
| Mahler (2014)[100] | 2010-2012 | 3 European countries | 38878 | 13957 | 35.9 | NA | 8278 | 21.3 |
| Hosteing (2014)[101] | 2010-2012 | France | 7874 | NA | NA | NA | NA | NA |
| Aerts (2014)[102] | 2010-2013 | Belgium | 8680 | NA | NA | NA | NA | NA |
| Scherrer (2014)[103] | 2009-2012 | Brazil | 359 | 45 | 12.5 | NA | 63 | 17.5 |
| De Unamuno (2014)[104] | 1980-2013 | Spain | 9109 | 3403 | 37.36 | NA | 1866 | 20.49 |
| Schwensen (2014)[105] | 2009-2012 | Denmark | 6744 | 2282 | 33.84 | NA | 3209 | 47.58 |
| Malinauskiene (2014)[106] | 2010-2012 | Sweden and Lithuania | 642 | 73 | 11.37 | NA | 274 | 42.68 |
| Lundov (2013)[107] | 2010-2012 | Denmark | 7981 | NA | NA | NA | NA | NA |
| Chow (2013)[108] | 1993-2006 | Australia | 6845 | 2856 | 41.72 | NA | NA | NA |
| Uter (2012)[109] | 2007-2008 | 11 European countries | 25181 | NA | NA | NA | NA | NA |
| Maio (2012)[110] | 2007-2009 | Portugal | 629 | 343 | 54.53 | NA | NA | NA |
| Lee (2012)[111] | 2010-2011 | South Korea | 584 | 104 | 17.9 | 35.02±12.23 | NA | NA |
| Uter (2012)[112] | 1992-2010 | 3 European countries | 171874 | NA | NA | NA | NA | NA |
| Schnuch (2011)[113] | 1996-2009 | 3 European countries | NA | NA | NA | NA | NA | NA |
| Ackermann (2011)[114] | 2006-2008 | Finland | 10821 | NA | NA | NA | NA | NA |
| Toledo (2011)[115] | 2005-2009 | Spain | 111 | 41 | 36.94 | 12±3.5 | 58 | 52.25 |
| **Abbreviations:** NA, Not Applicable; n, number; SD, standard deviation. | | | | | | | | |

| **Supplementary Table 3:** Appraisal tool for Cross-Sectional Studies (AXIS) assessment of included studies | | | | | | | | | | | | | | | | | | | | |
| --- | --- | --- | --- | --- | --- | --- | --- | --- | --- | --- | --- | --- | --- | --- | --- | --- | --- | --- | --- | --- |
|  | **Introduction** | **Methods** | | | | | | | | | | **Results** | | | | | **Discussion** | | **Other** | |
| **Reference** | **Q1** | **Q2** | **Q3** | **Q4** | **Q5** | **Q6** | **Q7** | **Q8** | **Q9** | **Q10** | **Q11** | **Q12** | **Q13** | **Q14** | **Q15** | **Q16** | **Q17** | **Q18** | **Q19** | **Q20** |
| Bonamonte (2025)[1] | 1 | 1 | 1 | 1 | 1 | 1 | 3 | 1 | 1 | 1 | 1 | 1 | 2 | 3 | 1 | 1 | 1 | 1 | 2 | 1 |
| Belluco (2024)[2] | 1 | 1 | 1 | 1 | 1 | 1 | 3 | 1 | 1 | 1 | 1 | 1 | 2 | 3 | 1 | 1 | 1 | 1 | 2 | 1 |
| Schwensen (2024)[3] | 1 | 1 | 1 | 1 | 1 | 1 | 3 | 1 | 1 | 1 | 1 | 1 | 2 | 3 | 1 | 1 | 1 | 2 | 3 | 3 |
| Lee (2024)[4] | 1 | 1 | 1 | 1 | 1 | 1 | 3 | 1 | 1 | 1 | 1 | 1 | 2 | 3 | 1 | 1 | 1 | 1 | 3 | 3 |
| Tous-Romero (2024)[5] | 1 | 1 | 1 | 1 | 1 | 1 | 3 | 1 | 1 | 1 | 1 | 1 | 2 | 3 | 1 | 1 | 1 | 2 | 2 | 1 |
| Kalay Yildizhan (2024)[6] | 1 | 1 | 1 | 1 | 1 | 1 | 3 | 1 | 1 | 1 | 1 | 1 | 2 | 3 | 1 | 1 | 1 | 1 | 2 | 1 |
| Arora (2024)[7] | 1 | 1 | 1 | 1 | 1 | 1 | 3 | 1 | 1 | 1 | 1 | 1 | 2 | 3 | 1 | 1 | 1 | 1 | 2 | 3 |
| Chicharro (2024)[8] | 1 | 1 | 1 | 1 | 1 | 1 | 3 | 1 | 1 | 1 | 1 | 1 | 2 | 3 | 1 | 1 | 1 | 1 | 2 | 1 |
| Reeder (2024)[9] | 1 | 1 | 1 | 1 | 1 | 1 | 3 | 1 | 1 | 1 | 1 | 1 | 2 | 3 | 1 | 1 | 1 | 1 | 3 | 3 |
| Slodownik (2024)[10] | 1 | 1 | 1 | 1 | 1 | 1 | 3 | 1 | 1 | 1 | 1 | 1 | 2 | 3 | 1 | 1 | 1 | 2 | 2 | 3 |
| Boonchai (2023)[11] | 1 | 1 | 1 | 1 | 1 | 1 | 3 | 1 | 1 | 1 | 1 | 1 | 2 | 3 | 1 | 1 | 1 | 1 | 2 | 3 |
| Akasya-Hillenbrand (2008)[12] | 1 | 1 | 1 | 1 | 1 | 1 | 3 | 1 | 1 | 1 | 1 | 1 | 2 | 3 | 1 | 1 | 1 | 1 | 2 | 3 |
| Dickel (2002)[13] | 1 | 1 | 1 | 1 | 1 | 1 | 3 | 1 | 1 | 1 | 1 | 1 | 2 | 3 | 1 | 1 | 1 | 1 | 2 | 3 |
| Sürgün (2024)[14] | 1 | 1 | 1 | 1 | 1 | 1 | 3 | 1 | 1 | 1 | 1 | 1 | 2 | 3 | 1 | 1 | 1 | 1 | 2 | 1 |
| Kazan (2023)[15] | 1 | 1 | 1 | 1 | 1 | 1 | 3 | 1 | 1 | 1 | 1 | 1 | 2 | 3 | 1 | 1 | 1 | 1 | 2 | 1 |
| Belluco (2023)[16] | 1 | 1 | 1 | 1 | 1 | 1 | 3 | 1 | 1 | 1 | 1 | 1 | 2 | 3 | 1 | 1 | 1 | 1 | 2 | 1 |
| Fernández (2023)[17] | 1 | 1 | 1 | 1 | 1 | 1 | 3 | 1 | 1 | 1 | 1 | 1 | 2 | 3 | 1 | 1 | 1 | 1 | 2 | 3 |
| Kaur (2023)[18] | 1 | 1 | 1 | 1 | 1 | 1 | 3 | 1 | 1 | 1 | 1 | 1 | 2 | 3 | 1 | 1 | 1 | 1 | 2 | 3 |
| Geier (2023)[19] | 1 | 1 | 1 | 1 | 1 | 1 | 3 | 1 | 1 | 1 | 1 | 1 | 2 | 3 | 1 | 1 | 1 | 1 | 2 | 3 |
| Puangpet (2023)[20] | 1 | 1 | 1 | 1 | 1 | 1 | 3 | 1 | 1 | 1 | 1 | 1 | 2 | 3 | 1 | 1 | 1 | 1 | 3 | 3 |
| Kakşi (2023)[21] | 1 | 1 | 1 | 1 | 1 | 1 | 3 | 1 | 1 | 1 | 1 | 1 | 2 | 3 | 1 | 1 | 1 | 1 | 2 | 1 |
| Németh (2022)[22] | 1 | 1 | 1 | 1 | 1 | 1 | 3 | 1 | 1 | 1 | 1 | 1 | 2 | 3 | 1 | 1 | 1 | 1 | 2 | 1 |
| Quintana (2022)[23] | 1 | 1 | 1 | 1 | 1 | 1 | 3 | 1 | 1 | 1 | 1 | 1 | 2 | 3 | 1 | 1 | 1 | 1 | 2 | 3 |
| Németh (2022)[24] | 1 | 1 | 1 | 1 | 1 | 1 | 3 | 1 | 1 | 1 | 1 | 1 | 2 | 3 | 1 | 1 | 1 | 1 | 2 | 3 |
| Uter (2022)[25] | 1 | 1 | 1 | 1 | 1 | 1 | 3 | 1 | 1 | 1 | 1 | 1 | 2 | 3 | 1 | 1 | 1 | 1 | 3 | 1 |
| Bauer (2023)[26] | 1 | 1 | 1 | 1 | 1 | 1 | 3 | 1 | 1 | 1 | 1 | 1 | 2 | 3 | 1 | 1 | 1 | 1 | 3 | 3 |
| Wee (2022)[27] | 1 | 1 | 1 | 1 | 1 | 1 | 3 | 1 | 1 | 1 | 1 | 1 | 2 | 3 | 1 | 1 | 1 | 1 | 2 | 1 |
| Bonamonte (2022)[28] | 1 | 1 | 1 | 1 | 1 | 1 | 3 | 1 | 1 | 1 | 1 | 1 | 2 | 3 | 1 | 1 | 1 | 1 | 2 | 1 |
| Noë (2022)[29] | 1 | 1 | 1 | 1 | 1 | 1 | 3 | 1 | 1 | 1 | 1 | 1 | 2 | 3 | 1 | 1 | 1 | 1 | 2 | 1 |
| Uter (2022)[30] | 1 | 1 | 1 | 1 | 1 | 1 | 3 | 1 | 1 | 1 | 1 | 1 | 2 | 3 | 1 | 1 | 1 | 2 | 3 | 1 |
| Rosenberg-Bezalel (2022)[31] | 1 | 1 | 1 | 1 | 1 | 1 | 3 | 1 | 1 | 1 | 1 | 1 | 2 | 3 | 1 | 1 | 1 | 1 | 3 | 1 |
| Silverberg (2022)[32] | 1 | 1 | 1 | 1 | 1 | 1 | 3 | 1 | 1 | 1 | 1 | 1 | 2 | 3 | 1 | 1 | 1 | 1 | 3 | 3 |
| Bizjak (2022)[33] | 1 | 1 | 1 | 1 | 1 | 1 | 3 | 1 | 1 | 1 | 1 | 1 | 2 | 3 | 1 | 1 | 1 | 1 | 3 | 3 |
| Andernord (2022)[34] | 1 | 1 | 1 | 1 | 1 | 1 | 3 | 1 | 1 | 1 | 1 | 1 | 2 | 3 | 1 | 1 | 1 | 1 | 3 | 1 |
| Uter (2021)[35] | 1 | 1 | 1 | 1 | 1 | 1 | 3 | 1 | 1 | 1 | 1 | 1 | 2 | 3 | 1 | 1 | 1 | 2 | 3 | 3 |
| Hernández-Fernández (2021)[36] | 1 | 1 | 1 | 1 | 1 | 1 | 3 | 1 | 1 | 1 | 1 | 1 | 2 | 3 | 1 | 1 | 1 | 1 | 2 | 1 |
| Atwater (2021)[37] | 1 | 1 | 1 | 1 | 1 | 1 | 3 | 1 | 1 | 1 | 1 | 1 | 2 | 3 | 1 | 1 | 1 | 1 | 3 | 3 |
| Boyvat (2021)[38] | 1 | 1 | 1 | 1 | 1 | 1 | 3 | 1 | 1 | 1 | 1 | 1 | 2 | 3 | 1 | 1 | 1 | 1 | 2 | 1 |
| Özkaya (2021)[39] | 1 | 1 | 1 | 1 | 1 | 1 | 3 | 1 | 1 | 1 | 1 | 1 | 2 | 3 | 1 | 1 | 1 | 1 | 2 | 1 |
| Havmose (2021)[40] | 1 | 1 | 1 | 1 | 1 | 1 | 3 | 1 | 1 | 1 | 1 | 1 | 2 | 3 | 1 | 1 | 1 | 1 | 2 | 3 |
| Yilmaz (2021)[41] | 1 | 1 | 1 | 1 | 1 | 1 | 3 | 1 | 1 | 1 | 1 | 1 | 2 | 3 | 1 | 1 | 1 | 1 | 2 | 1 |
| Woodruff (2022)[42] | 1 | 1 | 1 | 1 | 1 | 1 | 3 | 1 | 1 | 1 | 1 | 1 | 2 | 3 | 1 | 1 | 1 | 1 | 2 | 1 |
| Sukakul (2021)[43] | 1 | 1 | 1 | 1 | 1 | 1 | 3 | 1 | 1 | 1 | 1 | 1 | 2 | 3 | 1 | 1 | 1 | 2 | 2 | 3 |
| Isaksson (2021)[44] | 1 | 1 | 1 | 1 | 1 | 1 | 3 | 1 | 1 | 1 | 1 | 1 | 2 | 3 | 1 | 1 | 1 | 1 | 2 | 3 |
| Silverberg (2021)[45] | 1 | 1 | 1 | 1 | 1 | 1 | 3 | 1 | 1 | 1 | 1 | 1 | 2 | 3 | 1 | 1 | 1 | 1 | 3 | 1 |
| Atwater (2021)[46] | 1 | 1 | 1 | 1 | 1 | 1 | 3 | 1 | 1 | 1 | 1 | 1 | 2 | 3 | 1 | 1 | 1 | 1 | 3 | 1 |
| Lin (2021)[47] | 1 | 1 | 1 | 1 | 1 | 1 | 3 | 1 | 1 | 1 | 1 | 1 | 2 | 3 | 1 | 1 | 1 | 1 | 2 | 3 |
| Boonchai (2021)[48] | 1 | 1 | 1 | 1 | 1 | 1 | 3 | 1 | 1 | 1 | 1 | 1 | 2 | 3 | 1 | 1 | 1 | 1 | 2 | 1 |
| Herman (2021)[49] | 1 | 1 | 1 | 1 | 1 | 1 | 3 | 1 | 1 | 1 | 1 | 1 | 2 | 3 | 1 | 1 | 1 | 1 | 2 | 1 |
| Koca (2021)[50] | 1 | 1 | 1 | 1 | 1 | 1 | 3 | 1 | 1 | 1 | 1 | 1 | 2 | 3 | 1 | 1 | 1 | 1 | 2 | 1 |
| Toncic (2020)[51] | 1 | 1 | 1 | 1 | 1 | 1 | 3 | 1 | 1 | 1 | 1 | 1 | 2 | 3 | 1 | 1 | 1 | 1 | 3 | 3 |
| Felmingham (2020)[52] | 1 | 1 | 1 | 1 | 1 | 1 | 3 | 1 | 1 | 1 | 1 | 1 | 2 | 3 | 1 | 1 | 1 | 1 | 3 | 1 |
| Uter (2020)[53] | 1 | 1 | 1 | 1 | 1 | 1 | 3 | 1 | 1 | 1 | 1 | 1 | 2 | 3 | 1 | 1 | 1 | 1 | 3 | 3 |
| Tam (2020)[54] | 1 | 1 | 1 | 1 | 1 | 1 | 3 | 1 | 1 | 1 | 1 | 1 | 2 | 3 | 1 | 1 | 1 | 1 | 2 | 1 |
| Silva (2020)[55] | 1 | 1 | 1 | 1 | 1 | 1 | 3 | 1 | 1 | 1 | 1 | 1 | 2 | 3 | 1 | 1 | 1 | 1 | 2 | 1 |
| Pónyai (2019)[56] | 1 | 1 | 1 | 1 | 1 | 1 | 3 | 1 | 1 | 1 | 1 | 1 | 2 | 3 | 1 | 1 | 1 | 2 | 2 | 3 |
| Warshaw (2021)[57] | 1 | 1 | 1 | 1 | 1 | 1 | 3 | 1 | 1 | 1 | 1 | 1 | 2 | 3 | 1 | 1 | 1 | 1 | 3 | 1 |
| Engfeldt (2020)[58] | 1 | 1 | 1 | 1 | 1 | 1 | 3 | 1 | 1 | 1 | 1 | 1 | 2 | 3 | 1 | 1 | 1 | 2 | 3 | 3 |
| Sukakul (2019)[59] | 1 | 1 | 1 | 1 | 1 | 1 | 3 | 1 | 1 | 1 | 1 | 1 | 2 | 3 | 1 | 1 | 1 | 2 | 2 | 1 |
| Dusefante (2019)[60] | 1 | 1 | 1 | 1 | 1 | 1 | 3 | 1 | 1 | 1 | 1 | 1 | 2 | 3 | 1 | 1 | 1 | 1 | 2 | 3 |
| Salman (2019)[61] | 1 | 1 | 1 | 1 | 1 | 1 | 3 | 1 | 1 | 1 | 1 | 1 | 2 | 3 | 1 | 1 | 1 | 2 | 2 | 1 |
| Boonchai (2019)[62] | 1 | 1 | 1 | 1 | 1 | 1 | 3 | 1 | 1 | 1 | 1 | 1 | 2 | 3 | 1 | 1 | 1 | 1 | 3 | 1 |
| Winayanuwattikun (2019)[63] | 1 | 1 | 1 | 1 | 1 | 1 | 3 | 1 | 1 | 1 | 1 | 1 | 2 | 3 | 1 | 1 | 1 | 2 | 2 | 1 |
| Huang (2019)[64] | 1 | 1 | 1 | 1 | 1 | 1 | 3 | 1 | 1 | 1 | 1 | 1 | 2 | 3 | 1 | 1 | 1 | 1 | 2 | 3 |
| Fourie (2019)[65] | 1 | 1 | 1 | 1 | 1 | 1 | 3 | 1 | 1 | 1 | 1 | 1 | 2 | 3 | 1 | 1 | 1 | 1 | 2 | 3 |
| Hadžavdić (2018)[66] | 1 | 1 | 1 | 1 | 1 | 1 | 3 | 1 | 1 | 1 | 1 | 1 | 2 | 3 | 1 | 1 | 1 | 2 | 3 | 3 |
| Marrero-Alemán (2018)[67] | 1 | 1 | 1 | 1 | 1 | 1 | 3 | 1 | 1 | 1 | 1 | 1 | 2 | 3 | 1 | 1 | 1 | 1 | 2 | 3 |
| Flury (2018)[68] | 1 | 1 | 1 | 1 | 1 | 1 | 3 | 1 | 1 | 1 | 1 | 1 | 2 | 3 | 1 | 1 | 1 | 2 | 2 | 3 |
| Warshaw (2018)[69] | 1 | 1 | 1 | 1 | 1 | 1 | 3 | 1 | 1 | 1 | 1 | 1 | 2 | 3 | 1 | 1 | 1 | 1 | 2 | 1 |
| Simonsen (2018)[70] | 1 | 1 | 1 | 1 | 1 | 1 | 3 | 1 | 1 | 1 | 1 | 1 | 2 | 3 | 1 | 1 | 1 | 1 | 2 | 1 |
| Aalto-Korte (2017)[71] | 1 | 1 | 1 | 1 | 1 | 1 | 3 | 1 | 1 | 1 | 1 | 1 | 2 | 3 | 1 | 1 | 1 | 2 | 2 | 3 |
| Uter (2017)[72] | 1 | 1 | 1 | 1 | 1 | 1 | 3 | 1 | 1 | 1 | 1 | 1 | 2 | 3 | 1 | 1 | 1 | 2 | 3 | 3 |
| Schwensen (2017)[73] | 1 | 1 | 1 | 1 | 1 | 1 | 3 | 1 | 1 | 1 | 1 | 1 | 2 | 3 | 1 | 1 | 1 | 1 | 2 | 1 |
| How (2017)[74] | 1 | 1 | 1 | 1 | 1 | 1 | 3 | 1 | 1 | 1 | 1 | 1 | 2 | 3 | 1 | 1 | 1 | 1 | 3 | 3 |
| Dararattanaroj (2017)[75] | 1 | 1 | 1 | 1 | 1 | 1 | 3 | 1 | 1 | 1 | 1 | 1 | 2 | 3 | 1 | 1 | 1 | 2 | 3 | 1 |
| Jacob (2017)[76] | 1 | 1 | 1 | 1 | 1 | 1 | 3 | 1 | 1 | 1 | 1 | 1 | 2 | 3 | 1 | 1 | 1 | 1 | 3 | 1 |
| Bouschon (2019)[77] | 1 | 1 | 1 | 1 | 1 | 1 | 3 | 1 | 1 | 1 | 1 | 2 | 2 | 3 | 1 | 1 | 1 | 2 | 3 | 3 |
| Gimenéz-Arnau (2017)[78] | 1 | 1 | 1 | 1 | 1 | 1 | 3 | 1 | 1 | 1 | 1 | 1 | 2 | 3 | 1 | 1 | 1 | 1 | 3 | 3 |
| Liao (2017)[79] | 1 | 1 | 1 | 1 | 1 | 1 | 3 | 1 | 1 | 1 | 1 | 2 | 2 | 3 | 1 | 1 | 1 | 1 | 2 | 1 |
| Hasan (2005)[80] | 1 | 1 | 1 | 1 | 1 | 1 | 3 | 1 | 1 | 1 | 1 | 2 | 2 | 3 | 1 | 1 | 1 | 2 | 2 | 3 |
| Fortina (2016)[81] | 1 | 1 | 1 | 1 | 1 | 1 | 3 | 1 | 1 | 1 | 1 | 1 | 2 | 3 | 1 | 1 | 1 | 1 | 3 | 3 |
| Mortazavi (2016)[82] | 1 | 1 | 1 | 1 | 1 | 1 | 3 | 1 | 1 | 1 | 1 | 1 | 2 | 3 | 1 | 1 | 1 | 1 | 3 | 1 |
| Smith (2016)[83] | 1 | 1 | 1 | 1 | 1 | 1 | 3 | 1 | 1 | 1 | 1 | 1 | 2 | 3 | 1 | 1 | 1 | 2 | 2 | 3 |
| Yu (2016)[84] | 1 | 1 | 1 | 1 | 1 | 1 | 3 | 1 | 1 | 1 | 1 | 1 | 2 | 3 | 1 | 1 | 1 | 2 | 3 | 1 |
| Linauskienė (2016)[85] | 1 | 1 | 1 | 1 | 1 | 1 | 3 | 1 | 1 | 1 | 1 | 1 | 2 | 3 | 1 | 1 | 1 | 1 | 2 | 3 |
| Zaragoza-Ninet (2016)[86] | 1 | 1 | 1 | 1 | 1 | 1 | 3 | 1 | 1 | 1 | 1 | 1 | 2 | 3 | 1 | 1 | 1 | 2 | 2 | 1 |
| Pontén (2016)[87] | 1 | 1 | 1 | 1 | 1 | 1 | 3 | 1 | 1 | 1 | 1 | 1 | 2 | 3 | 1 | 1 | 1 | 1 | 2 | 3 |
| Fortina (2015)[88] | 1 | 1 | 1 | 1 | 1 | 1 | 3 | 1 | 1 | 1 | 1 | 1 | 2 | 3 | 1 | 1 | 1 | 1 | 3 | 3 |
| Geier (2015)[89] | 1 | 1 | 1 | 1 | 1 | 1 | 3 | 1 | 1 | 1 | 1 | 1 | 2 | 3 | 1 | 1 | 1 | 2 | 3 | 3 |
| Akan (2015)[90] | 1 | 1 | 1 | 1 | 1 | 1 | 3 | 1 | 1 | 1 | 1 | 2 | 2 | 3 | 1 | 1 | 1 | 1 | 3 | 1 |
| Isaksson (2015)[91] | 1 | 1 | 1 | 1 | 1 | 1 | 3 | 1 | 1 | 1 | 1 | 2 | 2 | 3 | 1 | 1 | 1 | 2 | 3 | 3 |
| Jong (2007)[92] | 1 | 1 | 1 | 1 | 1 | 1 | 3 | 1 | 1 | 1 | 1 | 2 | 2 | 3 | 1 | 1 | 1 | 2 | 2 | 3 |
| Schnuch (2008)[93] | 1 | 1 | 2 | 1 | 1 | 1 | 2 | 1 | 1 | 1 | 3 | 2 | 1 | 1 | 1 | 1 | 1 | 1 | 2 | 3 |
| Lundov (2010)[94] | 1 | 1 | 1 | 1 | 1 | 1 | 3 | 1 | 1 | 1 | 1 | 1 | 2 | 3 | 1 | 1 | 1 | 1 | 2 | 1 |
| Fortina (2011)[95] | 1 | 1 | 1 | 1 | 1 | 1 | 3 | 1 | 1 | 1 | 1 | 1 | 2 | 3 | 1 | 1 | 1 | 1 | 2 | 1 |
| Fall (2015)[96] | 1 | 1 | 1 | 1 | 1 | 1 | 3 | 1 | 1 | 1 | 1 | 1 | 2 | 3 | 1 | 1 | 1 | 1 | 2 | 1 |
| Toholka (2015)[97] | 1 | 1 | 1 | 1 | 1 | 1 | 3 | 1 | 1 | 1 | 1 | 1 | 2 | 3 | 1 | 1 | 1 | 1 | 2 | 3 |
| Vauhkala (2015)[98] | 1 | 1 | 1 | 1 | 1 | 1 | 3 | 1 | 1 | 1 | 1 | 2 | 2 | 3 | 1 | 1 | 1 | 2 | 2 | 1 |
| Dinkloh (2015)[99] | 1 | 1 | 1 | 1 | 1 | 1 | 3 | 1 | 1 | 1 | 1 | 1 | 2 | 3 | 1 | 1 | 1 | 1 | 3 | 3 |
| Mahler (2014)[100] | 1 | 1 | 1 | 1 | 1 | 1 | 3 | 1 | 1 | 1 | 1 | 2 | 2 | 3 | 1 | 1 | 1 | 2 | 3 | 3 |
| Hosteing (2014)[101] | 1 | 1 | 1 | 1 | 1 | 1 | 3 | 1 | 1 | 1 | 1 | 2 | 2 | 3 | 1 | 1 | 1 | 1 | 3 | 3 |
| Aerts (2014)[102] | 1 | 1 | 1 | 1 | 1 | 1 | 3 | 1 | 1 | 1 | 1 | 1 | 2 | 3 | 1 | 1 | 1 | 1 | 2 | 3 |
| Scherrer (2014)[103] | 1 | 1 | 1 | 1 | 1 | 1 | 3 | 1 | 1 | 1 | 1 | 1 | 2 | 3 | 1 | 1 | 1 | 2 | 2 | 3 |
| De Unamuno (2014)[104] | 1 | 1 | 1 | 1 | 1 | 1 | 3 | 1 | 1 | 1 | 1 | 1 | 2 | 3 | 1 | 1 | 1 | 2 | 2 | 1 |
| Schwensen (2014)[105] | 1 | 1 | 1 | 1 | 1 | 1 | 3 | 1 | 1 | 1 | 1 | 1 | 2 | 3 | 1 | 1 | 1 | 1 | 2 | 1 |
| Malinauskiene (2014)[106] | 1 | 1 | 1 | 1 | 1 | 1 | 3 | 1 | 1 | 1 | 1 | 1 | 2 | 3 | 1 | 1 | 1 | 1 | 2 | 3 |
| Lundov (2013)[107] | 1 | 1 | 1 | 1 | 1 | 1 | 3 | 1 | 1 | 1 | 1 | 1 | 2 | 3 | 1 | 1 | 1 | 1 | 2 | 3 |
| Chow (2013)[108] | 1 | 1 | 1 | 1 | 1 | 1 | 3 | 1 | 1 | 1 | 1 | 1 | 2 | 3 | 1 | 1 | 1 | 1 | 3 | 1 |
| Uter (2012)[109] | 1 | 1 | 1 | 1 | 1 | 1 | 3 | 1 | 1 | 1 | 1 | 1 | 2 | 3 | 1 | 1 | 1 | 2 | 3 | 3 |
| Maio (2012)[110] | 1 | 1 | 1 | 1 | 1 | 1 | 3 | 1 | 1 | 1 | 1 | 1 | 2 | 3 | 1 | 1 | 1 | 2 | 2 | 3 |
| Lee (2012)[111] | 1 | 1 | 1 | 1 | 1 | 1 | 3 | 1 | 1 | 1 | 1 | 1 | 2 | 3 | 1 | 1 | 1 | 2 | 3 | 3 |
| Uter (2012)[112] | 1 | 1 | 1 | 1 | 1 | 1 | 3 | 1 | 1 | 1 | 1 | 1 | 2 | 3 | 1 | 1 | 1 | 2 | 3 | 3 |
| Schnuch (2011)[113] | 1 | 1 | 1 | 1 | 1 | 1 | 3 | 1 | 1 | 1 | 1 | 1 | 2 | 3 | 1 | 1 | 1 | 2 | 3 | 3 |
| Ackermann (2011)[114] | 1 | 1 | 1 | 1 | 1 | 1 | 3 | 1 | 1 | 1 | 1 | 1 | 2 | 3 | 1 | 1 | 1 | 1 | 2 | 3 |
| Toledo (2011)[115] | 1 | 1 | 1 | 1 | 1 | 1 | 3 | 1 | 1 | 1 | 1 | 1 | 2 | 3 | 1 | 1 | 1 | 1 | 2 | 3 |
| **Abbreviations:** Q, question; 1, yes; 2, no; 3, don’t know/not applicable. | | | | | | | | | | | | | | | | | | | | |

**Supplementary figure 1:** The Preferred Reporting Items for Systematic Reviews and Meta-analyses (PRISMA) flowchart


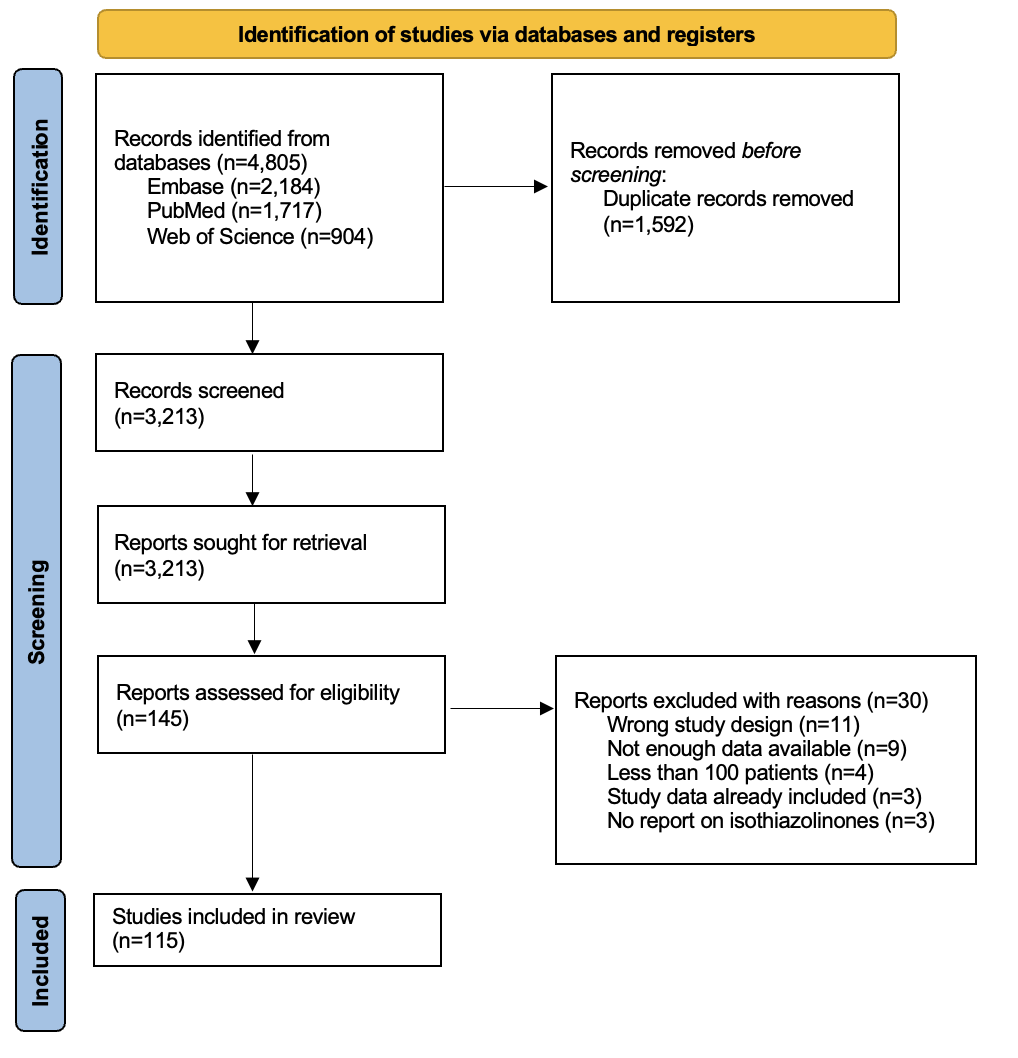


**Supplementary figure 2:** Funnel plot of contact allergy to Methylchloroisothiazolinone/methylisothiazolinone in all patients

**Supplementary figure 3:** Funnel plot of contact allergy to methylisothiazolinone in all patients

**Supplementary figure 4:** Funnel plot of contact allergy to benzisothiazolinone in all patients

**References**

1. Bonamonte D, De Marco A, Ciccarese G, Romita P, Giancaspro G, Ambrogio F, et al. Contact Allergy in Atopic Dermatitis and Psoriasis: A Retrospective Study. Diagnostics [Internet]. Multidisciplinary Digital Publishing Institute (MDPI); 2025 [cited 2025 Aug 29];15:766. https://doi.org/10.3390/DIAGNOSTICS15060766/S1

2. Belluco PES, Birolim MM, Ferreira MD, Belluco JEF, da Silva Maciel Azevedo F, da Mota Pinheiro B, et al. Prevalence and factors associated with sensitivity to methylisothiazolinone in individuals with suspected allergic contact dermatitis: A cross-sectional study. Rev Alerg Mex [Internet]. Colegio Mexicano de Inmunologia Clinica y Alergia A.C; 2025 [cited 2025 Aug 29];71:234–41. https://doi.org/10.29262/ram.v71i4.1418

3. Schwensen JFB, Uter W, Aerts O, Agner T, Brans R, Bruze M, et al. Current frequency of contact allergy to isothiazolinones (methyl-, benz- and octylisothiazolinone) across Europe. Contact Dermatitis [Internet]. John Wiley and Sons Inc; 2024 [cited 2025 Aug 29];91:271–7. https://doi.org/https://pubmed.ncbi.nlm.nih.gov/39021255/

4. Lee HK, Kennedy H. Methylisothiazolinone sensitisation in New Zealand is decreasing. Australasian Journal of Dermatology [Internet]. John Wiley & Sons, Ltd; 2024 [cited 2025 Aug 29];65:423–7. https://doi.org/10.1111/AJD.14301

5. Tous-Romero F, Borrego-Hernando L, García-Doval I, Mercader-García P, Silvester-Salvador JF, Sánchez-Gilo A, et al. Four-year Epidemiological Surveillance of the Spanish Registry of Research in Contact Dermatitis and Cutaneous Allergy: Current Situation and Trends. Actas Dermosifiliogr. Elsevier Doyma; 2024;115:331–40. https://doi.org/10.1016/J.AD.2023.10.039

6. Yildizhan IK, Boyvat A. The Contact Sensitivity of Turkish Children and Adolescents to European Baseline Series Allergens between 2013 and 2023. Dermatol Pract Concept [Internet]. Mattioli 1885; 2024 [cited 2025 Aug 29];14:e2024151. https://doi.org/10.5826/DPC.1402A151

7. Arora P, Brumley C, Hylwa S. Clinical relevance of doubtful reactions in patch testing: A single-centre retrospective study. Contact Dermatitis [Internet]. John Wiley & Sons, Ltd; 2024 [cited 2025 Aug 29];90:607–12. https://doi.org/10.1111/COD.14526

8. Chicharro P, Munera-Campos M, Zaragoza-Ninet V, Giménez-Arnau A, González-Pérez R, Miquel-Miquel FJ, et al. Allergic contact dermatitis in adults with and without atopic dermatitis: Evaluation of the Spanish Contact Dermatitis Registry (REIDAC). Contact Dermatitis [Internet]. John Wiley and Sons Inc; 2024 [cited 2025 Aug 29];91:404–11. https://doi.org/10.1111/COD.14672

9. Reeder MJ, Zhang D, Aravamuthan SR, Warshaw EM, DeKoven JG, Silverberg JI, et al. More than just methylisothiazolinone: Retrospective analysis of patients with isothiazolinone allergy in North America, 2017-2020. J Am Acad Dermatol [Internet]. Elsevier Inc.; 2024 [cited 2025 Aug 29];90:319–27. https://doi.org/10.1016/j.jaad.2023.10.032

10. Slodownik D, Bar J, Daniely D. Trends in contact sensitization, results, and implications from a contact dermatitis clinic in Israel. Contact Dermatitis [Internet]. John Wiley & Sons, Ltd; 2024 [cited 2025 Aug 29];90:556–65. https://doi.org/10.1111/COD.14524

11. Boonchai W, Likittanasombat S, Viriyaskultorn N, Kanokrungsee S. Gender differences in allergic contact dermatitis to common allergens. Contact Dermatitis [Internet]. John Wiley and Sons Inc; 2024 [cited 2025 Aug 29];90:458–65. https://doi.org/10.1111/COD.14479

12. Akasya-Hillenbrand E, Özkaya-Bayazit E. Patch test results in 542 patients with suspected contact dermatitis in Turkey. Contact Dermatitis [Internet]. John Wiley & Sons, Ltd; 2002 [cited 2025 Aug 29];46:17–23. https://doi.org/10.1034/J.1600-0536.2002.460104.X

13. Dickel H, Kuss O, Schmidt A, Diepgen TL. Occupational relevance of positive standard patch-test results in employed persons with an initial report of an occupational skin disease. Int Arch Occup Environ Health [Internet]. Springer; 2002 [cited 2025 Aug 29];75:423–34. https://doi.org/10.1007/S00420-002-0328-2

14. Sürgün E, Üniversitesi A, Fakültesi T, Sina Hastanesi Yerles I, Blok KB. Increased rates of contact allergy to selected preservatives in patients with allergic contact dermatitis in Turkey. Contact Dermatitis [Internet]. John Wiley & Sons, Ltd; 2024 [cited 2025 Aug 29];90:110–5. https://doi.org/10.1111/COD.14435

15. Kazan D, Odyakmaz-Demirsoy E, Kiran R, Şikar-Aktürk A, Sayman N, Bayramgürler D. Methyl(chloro)isothiazolinone contact allergy: a monocentric experience from Turkey. Cutan Ocul Toxicol [Internet]. Taylor and Francis Ltd.; 2023 [cited 2025 Aug 29];42:97–102. https://doi.org/10.1080/15569527.2023.2217244

16. Belluco PES, Giavina-Bianchi P, Belluco RZF, Novaes MRCG, Reis CMS. Prospective study of consecutive patch testing in patients with contact dermatitis using an adapted Latin American baseline series. Eur Ann Allergy Clin Immunol [Internet]. EDRA S.p.A; 2023 [cited 2025 Aug 29];55:235–42. https://doi.org/10.23822/EURANNACI.1764-1489.250,

17. Hernández Fernández CP, Borrego L, Mercader García P, Giménez Arnau AM, Sánchez Pérez J, Silvestre Salvador JF, et al. Sensitization to isothiazolinones in the Spanish Contact Dermatitis Registry (REIDAC): 2019–2021 epidemiological situation. Contact Dermatitis [Internet]. John Wiley & Sons, Ltd; 2023 [cited 2025 Aug 29];88:212–9. https://doi.org/10.1111/COD.14251

18. Kaur A, Nixon R, Palmer A. Occupational allergic contact dermatitis: are there emerging allergens? [Internet]. 2023 [cited 2025 Aug 29]. https://journals.co.za/doi/10.10520/ejc-caci-v36-n2-a5. Accessed 29 Aug 2025

19. Geier J, Brans R, Weisshaar E, Wagner N, Szliska C, Heratizadeh A, et al. Contact sensitization to benzisothiazolinone: IVDK-data of the years 2002 to 2021. Contact Dermatitis [Internet]. John Wiley and Sons Inc; 2023 [cited 2025 Aug 29];88:446–55. https://doi.org/10.1111/COD.14300

20. Puangpet P, Boonpuen N, Saipornchai K, Poompakdeepan P, Suchaoin R, McFadden J. Patch Testing of Thai Children with Eczema. Siriraj Med J [Internet]. Faculty of Medicine Siriraj Hospital, Mahidol University; 2023 [cited 2025 Aug 29];75:70–5. https://doi.org/10.33192/SMJ.V75I2.260740

21. Kakşi SA, Kahraman FC, Akdeniz N, Özen T. Results of the patch tests with European baseline series in children: Five years of experience from a single center in Turkey and a review of the literature. J Cosmet Dermatol [Internet]. John Wiley and Sons Inc; 2023 [cited 2025 Aug 29];22:1071–6. https://doi.org/10.1111/JOCD.15531

22. Németh D, Pónyai G. Contact Allergy in the Elderly: A Study of 600 Patients. [Internet]. MDPI; 2022 [cited 2025 Aug 29];12:1228. https://doi.org/10.3390/LIFE12081228

23. Roque Quintana B, Falcón Hernández A, Sagrera Guedes A, Borrego L. Contact Dermatitis to Allergens in the Spanish Standard Series: Patch Test Findings in the South of Gran Canaria. Actas Dermosifiliogr. Elsevier Doyma; 2022;113:555–62. https://doi.org/10.1016/J.AD.2022.02.027

24. Németh D, Temesvári E, Holló P, Pónyai G. Preservative Contact Hypersensitivity among Adult Atopic Dermatitis Patients. [Internet]. MDPI; 2022 [cited 2025 Aug 29];12:715. https://doi.org/10.3390/LIFE12050715

25. Uter W, Wilkinson SM, Aerts O, Bauer A, Borrego L, Buhl T, et al. European patch test results with audit allergens as candidates for inclusion in the European Baseline Series, 2019/20: Joint results of the ESSCAA and the EBSB working groups of the ESCD, and the GEIDACC. Contact Dermatitis [Internet]. John Wiley and Sons Inc; 2022 [cited 2025 Aug 29];86:379–89. https://doi.org/10.1111/COD.14059

26. Bauer A, Pesonen M, Brans R, Caroppo F, Dickel H, Dugonik A, et al. Occupational contact allergy: The European perspective–Analysis of patch test data from ESSCA between 2011 and 2020. Contact Dermatitis [Internet]. John Wiley and Sons Inc; 2023 [cited 2025 Aug 29];88:263–74. https://doi.org/10.1111/COD.14280

27. Wee C, Tan CH, Zhao X, Yew YW, Goon A. Pattern of contact sensitization in patients with and without atopic dermatitis in an Asian dermatology center. Contact Dermatitis [Internet]. John Wiley and Sons Inc; 2022 [cited 2025 Aug 29];86:398–403. https://doi.org/10.1111/COD.14068

28. Bonamonte D, Hansel K, Romita P, Fortina AB, Girolomoni G, Fabbrocini G, et al. Contact allergy in children with and without atopic dermatitis: An Italian multicentre study. Contact Dermatitis [Internet]. John Wiley & Sons, Ltd; 2022 [cited 2025 Aug 29];87:265–72. https://doi.org/10.1111/COD.14130

29. Noë E, Huygens S, Morren MA, Garmyn M, Goossens A, Gilissen L. Contact allergy in a paediatric population observed in a tertiary referral centre in Belgium. Contact Dermatitis. John Wiley and Sons Inc; 2022;86:3–8. https://doi.org/10.1111/COD.13975

30. Uter W, Wilkinson SM, Aerts O, Bauer A, Borrego L, Brans R, et al. Patch test results with the European baseline series, 2019/20—Joint European results of the ESSCA and the EBS working groups of the ESCD, and the GEIDAC. Contact Dermatitis [Internet]. John Wiley and Sons Inc; 2022 [cited 2025 Aug 29];87:343–55. https://doi.org/10.1111/COD.14170

31. Rosenberg-Bezalel S, Elbirt D, Mahlab-Guri K. Patch Testing in an Allergy Clinic: Real-world Experience [Internet]. 2022 [cited 2025 Aug 29]. https://www.ima.org.il/MedicineIMAJ/viewarticle.aspx?year=2022&month=10&page=649. Accessed 29 Aug 2025

32. Silverberg JI, Hou A, Warshaw EM, DeKoven JG, Maibach HI, Belsito D V., et al. Age-related differences in patch testing results among children: Analysis of North American Contact Dermatitis Group Data, 2001-2018. J Am Acad Dermatol [Internet]. Elsevier Inc.; 2022 [cited 2025 Aug 29];86:818–26. https://doi.org/10.1016/j.jaad.2021.07.030

33. Bizjak M, Adamič K, Bajrovič N, Eržen R, Jošt M, Kopač P, et al. Patch testing with the European baseline series and 10 added allergens: Single-centre study of 748 patients. Contact Dermatitis [Internet]. John Wiley & Sons, Ltd; 2022 [cited 2025 Aug 29];87:439–46. https://doi.org/10.1111/COD.14178

34. Andernord D, Bruze M, Bryngelsson IL, Bråred Christensson J, Glas B, Hagvall L, et al. Contact allergy to haptens in the Swedish baseline series: Results from the Swedish Patch Test Register (2010 to 2017). Contact Dermatitis [Internet]. John Wiley & Sons, Ltd; 2022 [cited 2025 Aug 29];86:175–88. https://doi.org/10.1111/COD.13996

35. Uter W, Zetzmann A, Ofenloch R, Schliemann S, Bruze M, Gonçalo M, et al. Prevalence of contact allergies in the population compared to a tertiary referral patch test clinic in Jena/Germany. Contact Dermatitis [Internet]. John Wiley & Sons, Ltd; 2021 [cited 2025 Aug 29];85:563–71. https://doi.org/10.1111/COD.13923

36. Hernández-Fernández CP, Mercader-García P, Silvestre Salvador JF, Sánchez Pérez J, Fernández Redondo V, Miquel Miquel FJ, et al. Candidate allergens for inclusion in the Spanish Standard Series based on data from the Spanish Contact Dermatitis Registry. Actas Dermo-Sifiliográficas (English Edition) [Internet]. Elsevier Doyma; 2021 [cited 2025 Aug 29];112:798–805. https://doi.org/10.1016/J.ADENGL.2021.07.013

37. Atwater AR, Petty AJ, Liu B, Green CL, Silverberg JI, DeKoven JG, et al. Contact dermatitis associated with preservatives: Retrospective analysis of North American Contact Dermatitis Group data, 1994 through 2016. J Am Acad Dermatol [Internet]. Mosby Inc.; 2021 [cited 2025 Aug 29];84:965–76. https://doi.org/10.1016/j.jaad.2020.07.059

38. Boyvat A, Kalay Yildizhan I. Patch test results of the European baseline series among 1309 patients in Turkey between 2013 and 2019. Contact Dermatitis [Internet]. John Wiley & Sons, Ltd; 2021 [cited 2025 Aug 29];84:15–23. https://doi.org/10.1111/COD.13653

39. Özkaya E, Kılıç Sayar S, Babuna Kobaner G, Pehlivan G. Methylchloroisothiazolinone/methylisothiazolinone and methylisothiazolinone contact allergy: A 24-year, single-center, retrospective cohort study from Turkey. Contact Dermatitis [Internet]. John Wiley & Sons, Ltd; 2021 [cited 2025 Aug 29];84:24–33. https://doi.org/10.1111/COD.13656

40. Havmose M, Thyssen JP, Zachariae C, Menné T, Johansen JD. The epidemic of contact allergy to methylisothiazolinone—An analysis of Danish consecutive patients patch tested between 2005 and 2019. Contact Dermatitis [Internet]. Blackwell Publishing Ltd; 2021 [cited 2025 Aug 29];84:254–62. https://doi.org/10.1111/COD.13717

41. Yılmaz Z, Özkaya E. Patch test results in terms of the recently recommended allergens in children and adolescents: A retrospective cohort study over 22 years from Turkey. Contact Dermatitis [Internet]. John Wiley & Sons, Ltd; 2021 [cited 2025 Aug 29];85:198–210. https://doi.org/10.1111/COD.13842

42. Woodruff CM, Kollhoff A, Butler DC, Botto N. Update on Contact Sensitization in the Older Adult Population. Dermatitis [Internet]. Lippincott Williams and Wilkins; 2022 [cited 2025 Aug 29];33:122–8. https://doi.org/10.1097/DER.0000000000000763

43. Sukakul T, Limphoka P, Boonchai W. Methylchloroisothiazolinone and/or Methylisothiazolinone Contact Allergies in Thailand. Dermatitis [Internet]. Wolters Kluwer Health; 2021 [cited 2025 Aug 29];32:375–80. https://doi.org/10.1097/DER.0000000000000537

44. Isaksson M, Andersen KE, Elsner P, Goh CL, Gonçalo M, Goossens A, et al. Patch Testing with Methylchloroisothiazolinone/Methylisothiazolinone Using a New Diagnostic Mix - A Multicenter Study from the International Contact Dermatitis Research Group. Dermatitis [Internet]. Lippincott Williams and Wilkins; 2021 [cited 2025 Aug 29];32:220–4. https://doi.org/10.1097/DER.0000000000000657

45. Silverberg JI, Hou A, Warshaw EM, DeKoven JG, Maibach HI, Belsito D V., et al. Prevalence and Trend of Allergen Sensitization in Adults and Children with Atopic Dermatitis Referred for Patch Testing, North American Contact Dermatitis Group Data, 2001-2016. Journal of Allergy and Clinical Immunology: In Practice [Internet]. American Academy of Allergy, Asthma and Immunology; 2021 [cited 2025 Aug 29];9:2853-2866.e14. https://doi.org/10.1016/j.jaip.2021.03.028

46. Atwater AR, Ward JM, Liu B, Warshaw EM, Dekoven JG, Silverberg JI, et al. Contact Allergy in Canada Versus United States: Analysis of the North American Contact Dermatitis Group Data 2005-2016. Dermatitis [Internet]. Wolters Kluwer Health; 2021 [cited 2025 Aug 29];32:421–9. https://doi.org/10.1097/DER.0000000000000701

47. Lin PH, Tseng YH, Chu CY. Changing trends of contact allergens: A 40-year retrospective study from a referral centre in northern Taiwan. Contact Dermatitis [Internet]. Blackwell Publishing Ltd; 2021 [cited 2025 Aug 29];85:39–45. https://doi.org/10.1111/COD.13795

48. Boonchai W, Chaiyabutr C, Charoenpipatsin N, Sukakul T. Pediatric contact allergy: A comparative study with adults. Contact Dermatitis [Internet]. Blackwell Publishing Ltd; 2021 [cited 2025 Aug 29];84:34–40. https://doi.org/10.1111/COD.13672

49. Herman A, Aerts O, Jacobs MC, Scheers C, Gilissen L, Goossens A, et al. Evolution of methylisothiazolinone sensitization: A Belgian multicentric study from 2014 to 2019. Contact Dermatitis [Internet]. John Wiley & Sons, Ltd; 2021 [cited 2025 Aug 29];85:643–9. https://doi.org/10.1111/COD.13956

50. Koca R, Kocaturk E, Savk E, Baskan EB, Aydin F, Yalcin B, et al. Patch Test Results to European Baseline Series in Turkey: A Prospective and Multicenter Study. Dermatitis [Internet]. Wolters Kluwer Health; 2021 [cited 2025 Aug 29];32:397–405. https://doi.org/10.1097/DER.0000000000000631

51. Tončić RJ, Hadžavdić SL, Pustišek N, Kulišić SM, Švigir A. Contact Sensitivity in Patients with Atopic Dermatitis. ACTA DERMATOVENEROLOGICA CROATICA Acta Dermatovenerol Croat. 2020;28:197–203.

52. Felmingham C, Davenport R, Bala H, Palmer A, Nixon R. Allergic contact dermatitis in children and proposal for an Australian Paediatric Baseline Series. Australasian Journal of Dermatology [Internet]. John Wiley & Sons, Ltd; 2020 [cited 2025 Aug 29];61:33–8. https://doi.org/10.1111/AJD.13169

53. Uter W, Gefeller O, Mahler V, Geier J. Trends and current spectrum of contact allergy in Central Europe: results of the Information Network of Departments of Dermatology (IVDK) 2007–2018. British Journal of Dermatology [Internet]. Oxford Academic; 2020 [cited 2025 Aug 29];183:857–65. https://doi.org/10.1111/BJD.18946

54. Tam I, Schalock PC, González E, Yu J. Patch Testing Results from the Massachusetts General Hospital Contact Dermatitis Clinic, 2007-2016. Dermatitis [Internet]. Lippincott Williams and Wilkins; 2020 [cited 2025 Aug 29];31:202–8. https://doi.org/10.1097/DER.0000000000000593

55. Silva EA, Bosco MRM, Lozano RR, Latini ACP, Souza VNB de. High rate of sensitization to Kathon CG, detected by patch tests in patients with suspected allergic contact dermatitis. An Bras Dermatol [Internet]. Elsevier Espana S.L.; 2020 [cited 2025 Aug 29];95:194. https://doi.org/10.1016/J.ABD.2019.09.026

56. Pónyai G, Németh I, Nagy G, Fábos B, Irinyi B, Dinnyés M, et al. Methylchloroisothiazolinone/methylisothiazolinone and methylisothiazolinone hypersensitivity in 1122 patients: A national multicenter study organized by the Hungarian Contact Dermatitis Group. Contact Dermatitis [Internet]. John Wiley & Sons, Ltd; 2019 [cited 2025 Aug 29];81:467–9. https://doi.org/10.1111/COD.13370

57. Warshaw EM, Schlarbaum JP, Silverberg JI, DeKoven JG, Fransway AF, Taylor JS, et al. Contact dermatitis to personal care products is increasing (but different!) in males and females: North American Contact Dermatitis Group data, 1996-2016. J Am Acad Dermatol [Internet]. Elsevier Inc.; 2021 [cited 2025 Aug 29];85:1446–55. https://doi.org/10.1016/j.jaad.2020.10.003

58. Engfeldt M, Isaksson M, Bråred-Christensson J, Hagvall L, Matura M, Ryberg K, et al. Can patch testing with methylchloroisothiazolinone/methylisothiazolinone be optimized using a new diagnostic mix? – A multicenter study from the Swedish Contact Dermatitis Research Group. Contact Dermatitis [Internet]. John Wiley & Sons, Ltd; 2020 [cited 2025 Aug 29];82:283–9. https://doi.org/10.1111/COD.13483

59. Sukakul T, Chaweekulrat P, Limphoka P, Boonchai W. Changing trends of contact allergens in Thailand: A 12-year retrospective study. Contact Dermatitis [Internet]. Blackwell Publishing Ltd; 2019 [cited 2025 Aug 29];81:124–9. https://doi.org/10.1111/COD.13289

60. Dusefante A, Mauro M, Belloni Fortina A, Corradin MT, Larese Filon F. Contact allergy to methylchloroisothiazolinone/methylisothiazolinone in north-eastern Italy: a temporal trend from 1996 to 2016. Journal of the European Academy of Dermatology and Venereology [Internet]. Blackwell Publishing Ltd; 2019 [cited 2025 Aug 29];33:912–7. https://doi.org/10.1111/JDV.15453

61. Salman A. Methylchloroisothiazolinone and methylisothiazolinone contact allergy: A retrospective cohort study from a tertiary dermatology clinic in Turkey. Contact Dermatitis [Internet]. John Wiley & Sons, Ltd; 2019 [cited 2025 Aug 29];80:193–4. https://doi.org/10.1111/COD.13175

62. Boonchai W, Maneeprasopchoke P, Chaweekulrat P, Kasemsarn P. Associated factors of widespread pattern of dermatitis among patch test population: 12-Year retrospective study. Australasian Journal of Dermatology [Internet]. Blackwell Publishing; 2019 [cited 2025 Aug 29];60:e40–5. https://doi.org/10.1111/AJD.12903

63. Winayanuwattikun W, Boonchai W. Factors associated with multiple contact allergies in Thai dermatitis patients: A 10-year retrospective study. Contact Dermatitis [Internet]. Blackwell Publishing Ltd; 2019 [cited 2025 Aug 29];80:279–85. https://doi.org/10.1111/COD.13189

64. Huang YK, Wu YH, Lu PH, Tu ME. Contact allergy to preservatives in Taiwan between 1996 and 2015. Dermatologica Sinica [Internet]. Wolters Kluwer Medknow Publications; 2019 [cited 2025 Aug 29];37:123–8. https://doi.org/10.4103/DS.DS_21_18

65. Fourie A, Carman H, Singh T. Sensitisation to the preservatives methylchloroisothiazolinone/methylisothiazolinone and methylisothiazolinone. Current Allergy & Clinical Immunology. 2019;32.

66. Ljubojević Hadžavdić S, Uter W, Ilijanić Samošćanec M, Johansen JD. Methylisothiazolinone contact allergy in Croatia: Epidemiology and course of disease following patch testing. Contact Dermatitis [Internet]. John Wiley & Sons, Ltd; 2018 [cited 2025 Aug 29];79:162–7. https://doi.org/10.1111/COD.13028

67. Marrero-Alemán G, Saavedra Santana P, Liuti F, Hernández N, López-Jiménez E, Borrego L. The Role of Cleaning Products in Epidemic Allergic Contact Dermatitis to Methylchloroisothiazolinone/Methylisothiazolinone. Dermatitis [Internet]. Lippincott Williams and Wilkins; 2018 [cited 2025 Aug 29];29:77–80. https://doi.org/10.1097/DER.0000000000000352

68. Flury U, Palmer A, Nixon R. The methylisothiazolinone contact allergy epidemic in Australia. Contact Dermatitis [Internet]. Blackwell Publishing Ltd; 2018 [cited 2025 Aug 29];79:189–91. https://doi.org/10.1111/COD.13025

69. Warshaw EM, Goodier MC, Dekoven JG, Maibach HI, Taylor JS, Sasseville D, et al. Contact Dermatitis Associated with Skin Cleansers: Retrospective Analysis of North American Contact Dermatitis Group Data 2000-2014. Dermatitis [Internet]. Lippincott Williams and Wilkins; 2018 [cited 2025 Aug 29];29:32–42. https://doi.org/10.1097/DER.0000000000000330

70. Simonsen AB, Foss-Skiftesvik MH, Thyssen JP, Deleuran M, Mortz CG, Zachariae C, et al. Contact allergy in Danish children: Current trends. Contact Dermatitis [Internet]. Blackwell Publishing Ltd; 2018 [cited 2025 Aug 29];79:295–302. https://doi.org/10.1111/COD.13079

71. Aalto-Korte K, Suuronen K. Patterns of concomitant allergic reactions in patients suggest cross-sensitization between octylisothiazolinone and methylisothiazolinone. Contact Dermatitis [Internet]. John Wiley & Sons, Ltd; 2017 [cited 2025 Aug 29];77:385–9. https://doi.org/10.1111/COD.12855

72. Uter W, Amario-Hita JC, Balato A, Ballmer-Weber B, Bauer A, Belloni Fortina A, et al. European Surveillance System on Contact Allergies (ESSCA): results with the European baseline series, 2013/14. Journal of the European Academy of Dermatology and Venereology [Internet]. Blackwell Publishing Ltd; 2017 [cited 2025 Aug 29];31:1516–25. https://doi.org/10.1111/JDV.14423

73. Schwensen JF, Uter W, Bruze M, Svedman C, Goossens A, Wilkinson M, et al. The epidemic of methylisothiazolinone: a European prospective study. Contact Dermatitis [Internet]. Blackwell Publishing Ltd; 2017 [cited 2025 Aug 29];76:272–9. https://doi.org/10.1111/COD.12733

74. Nien How K, Moon Tang M, Kaur RA, Jagjit Singh P, Johar A. Contact sensitisation in adults: a 5-year retrospective review in hospital Kuala Lumpur.

75. Dararattanaroj W, Pootongkam S, Rojanawatsirivej N, Wongpiyabovorn J. Patterns and risk factors of causative contact allergens in Thai adult patients with contact dermatitis. Asian Pac J Allergy Immunol [Internet]. Allergy and Immunology Society of Thailand; 2017 [cited 2025 Aug 29];35:27–32. https://doi.org/10.12932/AP0757

76. Jacob SE, McGowan M, Silverberg NB, Pelletier JL, Fonacier L, Mousdicas N, et al. Pediatric Contact Dermatitis Registry Data on Contact Allergy in Children With Atopic Dermatitis. JAMA Dermatol [Internet]. American Medical Association; 2017 [cited 2025 Aug 29];153:765. https://doi.org/10.1001/JAMADERMATOL.2016.6136

77. Bouschon P, Waton J, Pereira B, Schmutz JL, Le Bouëdec MCF, D’Incan M. Methylisothiazolinone allergic contact dermatitis: Assessment of relapses in 139 patients after avoidance advice. Contact Dermatitis [Internet]. Blackwell Publishing Ltd; 2019 [cited 2025 Aug 29];80:304–10. https://doi.org/10.1111/COD.13221

78. Giménez-Arnau AM, Deza G, Bauer A, Johnston GA, Mahler V, Schuttelaar ML, et al. Contact allergy to preservatives: ESSCA* results with the baseline series, 2009–2012. Journal of the European Academy of Dermatology and Venereology [Internet]. Blackwell Publishing Ltd; 2017 [cited 2025 Aug 29];31:664–71. https://doi.org/10.1111/JDV.14063

79. Liao SL, Tseng YH, Chu CY. Contact allergy to methylisothiazolinone/methylchloroisothiazolinone: A retrospective case series in a referral center in northern Taiwan. Dermatologica Sinica [Internet]. No longer published by Elsevier; 2017 [cited 2025 Aug 29];35:201–5. https://doi.org/10.1016/J.DSI.2017.06.006

80. Hasan T, Rantanen T, Alanko K, Harvima RJ, Jolanki R, Kalimo K, et al. Patch test reactions to cosmetic allergens in 1995-1997 and 2000-2002 in Finland - A multicentre study. Contact Dermatitis [Internet]. John Wiley & Sons, Ltd; 2005 [cited 2025 Aug 29];53:40–5. https://doi.org/10.1111/J.0105-1873.2005.00630.x

81. Belloni Fortina A, Fontana E, Peserico A. Contact sensitization in children: A retrospective study of 2,614 children from a single center. Pediatr Dermatol [Internet]. Blackwell Publishing Inc.; 2016 [cited 2025 Aug 29];33:399–404. https://doi.org/10.1111/PDE.12873

82. Mortazavi H, Ehsani A, Sajjadi SS, Aghazadeh N, Arian E. Patch testing in Iranian children with allergic contact dermatitis. BMC Dermatol [Internet]. BioMed Central Ltd.; 2016 [cited 2025 Aug 29];16:10. https://doi.org/10.1186/S12895-016-0047-0

83. Smith VM, Clark SM, Wilkinson M. Allergic contact dermatitis in children: trends in allergens, 10 years on. A retrospective study of 500 children tested between 2005 and 2014 in one UK centre. Contact Dermatitis [Internet]. John Wiley & Sons, Ltd; 2016 [cited 2025 Aug 29];74:37–43. https://doi.org/10.1111/COD.12489

84. Yu SH, Sood A, Taylor JS. Patch Testing for Methylisothiazolinone and Methylchloroisothiazolinone-Methylisothiazolinone Contact Allergy. JAMA Dermatol [Internet]. American Medical Association; 2016 [cited 2025 Aug 29];152:67–72. https://doi.org/10.1001/JAMADERMATOL.2015.3606

85. Linauskienė K, Malinauskienė L, Blažienė A. Time trends of contact allergy to the European baseline series in Lithuania. Contact Dermatitis [Internet]. Blackwell Publishing Ltd; 2017 [cited 2025 Aug 29];76:350–6. https://doi.org/10.1111/COD.12726

86. Zaragoza-Ninet V, Blasco Encinas R, Vilata-Corell JJ, Pérez-Ferriols A, Sierra-Talamantes C, Esteve-Martínez A, et al. Allergic Contact Dermatitis Due to Cosmetics: A Clinical and Epidemiological Study in a Tertiary Hospital. Actas Dermo-Sifiliográficas (English Edition) [Internet]. Elsevier BV; 2016 [cited 2025 Aug 29];107:329–36. https://doi.org/10.1016/J.ADENGL.2016.02.022

87. Pontén A, Bruze M, Engfeldt M, Hauksson I, Isaksson M. Concomitant contact allergies to formaldehyde, methylchloroisothiazolinone/methylisothiazolinone, methylisothiazolinone, and fragrance mixes I and II. Contact Dermatitis [Internet]. Blackwell Publishing Ltd; 2016 [cited 2025 Aug 29];75:285–9. https://doi.org/10.1111/COD.12598

88. Belloni Fortina A, Cooper SM, Spiewak R, Fontana E, Schnuch A, Uter W. Patch test results in children and adolescents across Europe. Analysis of the ESSCA Network 2002-2010. Pediatric Allergy and Immunology [Internet]. John Wiley & Sons, Ltd; 2015 [cited 2025 Aug 29];26:446–55. https://doi.org/10.1111/PAI.12397

89. Geier J, Lessmann H, Schnuch A, Uter W. Concomitant reactivity to methylisothiazolinone, benzisothiazolinone, and octylisothiazolinone. International Network of Departments of Dermatology data, 2009–2013. Contact Dermatitis [Internet]. John Wiley & Sons, Ltd; 2015 [cited 2025 Aug 29];72:337–9. https://doi.org/10.1111/COD.12347

90. Akan A, Toyran M, Vezi̇r E, Azkur D, Kaya A, Erkoçoğlu M, et al. The patterns and clinical relevance of contact allergen sensitization in a pediatric population with atopic dermatitis. [cited 2025 Aug 29]; https://doi.org/10.3906/sag-1309-62

91. Isaksson M, Hauksson I, Hindsén M, Pontén A, Svedman C, Bruze M. Methylisothiazolinone Contact Allergy is Rising to Alarming Heights Also in Southern Sweden [Internet]. 2015 [cited 2025 Aug 29]. https://medicaljournalssweden.se/actadv/article/view/5604/8498. Accessed 29 Aug 2025

92. Jong CT, Statham BN, Green CM, King CM, Gawkrodger DJ, Sansom JE, et al. Contact sensitivity to preservatives in the UK, 2004-2005: Results of multicentre study. Contact Dermatitis [Internet]. John Wiley & Sons, Ltd; 2007 [cited 2025 Aug 29];57:165–8. https://doi.org/10.1111/J.1600-0536.2007.01181.x

93. Schnuch A, Uter W, Lessmann H, Geier J. Contact allergy to preservatives. Results of the information network of departments of dermatology (IVDK) 1996 to 2007. Allergo Journal [Internet]. Urban und Vogel GmbH; 2008 [cited 2025 Aug 29];17:631–8. https://doi.org/10.1007/BF03361953/

94. Lundov MD, Thyssen JP, Zachariae C, Johansen JD. Prevalence and cause of methylisothiazolinone contact allergy. Contact Dermatitis [Internet]. John Wiley & Sons, Ltd; 2010 [cited 2025 Aug 29];63:164–7. https://doi.org/10.1111/j.1600-0536.2010.01774.x

95. Belloni Fortina A, Romano I, Peserico A, Eichenfield LF. Contact sensitization in very young children. J Am Acad Dermatol [Internet]. Mosby; 2011 [cited 2025 Aug 29];65:772–9. https://doi.org/10.1016/J.JAAD.2010.07.030

96. Fall S, Bruze M, Isaksson M, Lidén C, Matura M, Stenberg B, et al. Contact allergy trends in Sweden – a retrospective comparison of patch test data from 1992, 2000, and 2009. Contact Dermatitis [Internet]. John Wiley & Sons, Ltd; 2015 [cited 2025 Aug 29];72:297–304. https://doi.org/10.1111/COD.12346

97. Toholka R, Wang YS, Tate B, Tam M, Cahill J, Palmer A, et al. The first Australian Baseline Series: Recommendations for patch testing in suspected contact dermatitis. Australasian Journal of Dermatology [Internet]. John Wiley & Sons, Ltd; 2015 [cited 2025 Aug 29];56:107–15. https://doi.org/10.1111/AJD.12186

98. Vauhkala AR, Pesonen M, Suomela S, Kuuliala O, Suuronen K, Aalto-Korte K. Occupational contact allergy to methylchloroisothiazolinone/methylisothiazolinone and methylisothiazolinone. Contact Dermatitis [Internet]. John Wiley & Sons, Ltd; 2015 [cited 2025 Aug 29];73:150–6. https://doi.org/10.1111/COD.12413

99. Dinkloh A, Worm M, Geier J, Schnuch A, Wollenberg A. Contact sensitization in patients with suspected cosmetic intolerance: Results of the IVDK 2006-2011. Journal of the European Academy of Dermatology and Venereology [Internet]. John Wiley & Sons, Ltd; 2015 [cited 2025 Aug 29];29:1071–81. https://doi.org/10.1111/JDV.12750

100. Mahler V, Geier J, Schnuch A. Current trends in patch testing - New data from the German Contact Dermatitis Research Group (DKG) and the Information Network of Departments of Dermatology (IVDK). JDDG - Journal of the German Society of Dermatology [Internet]. Wiley-VCH Verlag; 2014 [cited 2025 Aug 29];12:583–92. https://doi.org/10.1111/DDG.12371

101. Hosteing S, Meyer N, Waton J, Barbaud A, Bourrain JL, Raison-Peyron N, et al. Outbreak of contact sensitization to methylisothiazolinone: An analysis of French data from the REVIDAL-GERDA network. Contact Dermatitis [Internet]. Blackwell Publishing Ltd; 2014 [cited 2025 Aug 29];70:262–9. https://doi.org/10.1111/COD.12207

102. Aerts O, Baeck M, Constandt L, Dezfoulian B, Jacobs MC, Kerre S, et al. The dramatic increase in the rate of methylisothiazolinone contact allergy in Belgium: A multicentre study. Contact Dermatitis [Internet]. Blackwell Publishing Ltd; 2014 [cited 2025 Aug 29];71:41–8. https://doi.org/10.1111/COD.12249

103. Scherrer MAR, Rocha VB. Increasing trend of sensitization to Methylchloroisothiazolinone/methylisothiazolinone (MCI/MI). An Bras Dermatol [Internet]. Sociedade Brasileira de Dermatologia; 2014 [cited 2025 Aug 29];89:527. https://doi.org/10.1590/ABD1806-4841.20142852

104. De Unamuno B, Zaragoza Ninet V, Sierra C, De La Cuadra J. Descriptive Study of Sensitization to Methylchloroisothiazolinone and Methylisothiazolinone in a Skin Allergy Unit. Actas Dermosifiliogr [Internet]. Elsevier; 2014 [cited 2025 Aug 29];105:854–9. https://doi.org/10.1016/J.ADENGL.2014.09.009

105. Schwensen JF, Menné T, Andersen KE, Sommerlund M, Johansen JD. Occupations at risk of developing contact allergy to isothiazolinones in Danish contact dermatitis patients: Results from a Danish multicentre study (2009-2012). Contact Dermatitis [Internet]. Blackwell Publishing Ltd; 2014 [cited 2025 Aug 29];71:295–302. https://doi.org/10.1111/COD.12286

106. L M, M B. Patch Testing with the Swedish Baseline Series in Two Countries. J Clin Exp Dermatol Res [Internet]. Longdom Publishing S.L; 2015 [cited 2025 Aug 29];6:1–6. https://doi.org/10.4172/2155-9554.10000299

107. Lundov MD, Opstrup MS, Johansen JD. Methylisothiazolinone contact allergy – a growing epidemic. Contact Dermatitis [Internet]. John Wiley & Sons, Ltd; 2013 [cited 2025 Aug 29];69:271–5. https://doi.org/10.1111/COD.12149

108. Chow ET, Avolio AM, Lee A, Nixon R. Frequency of positive patch test reactions to preservatives: The Australian experience. Australasian Journal of Dermatology [Internet]. John Wiley & Sons, Ltd; 2013 [cited 2025 Aug 29];54:31–5. https://doi.org/10.1111/J.1440-0960.2012.00958.x.

109. Uter W, Aberer W, Armario-Hita JC, Fernandez-Vozmediano JM, Ayala F, Balato A, et al. Current patch test results with the European baseline series and extensions to it from the ‘European Surveillance System on Contact Allergy’ network, 2007–2008. Contact Dermatitis [Internet]. John Wiley & Sons, Ltd; 2012 [cited 2025 Aug 29];67:9–19. https://doi.org/10.1111/J.1600-0536.2012.02070.X

110. Maio P, Carvalho R, Amaro C, Santos R, Cardoso J. Contact allergy to methylchoroisothiazolinone/methylisothiazolinone (MCI/MI): Findings from a Contact Dermatitis Unit. Cutan Ocul Toxicol [Internet]. Taylor & Francis; 2012 [cited 2025 Aug 29];31:151–3. https://doi.org/10.3109/15569527.2011.627522

111. Lee SS, Hong DK, Jeong NJ, Lee JH, Choi YS, Lee AY, et al. Multicenter study of preservative sensitivity in patients with suspected cosmetic contact dermatitis in Korea. J Dermatol [Internet]. John Wiley & Sons, Ltd; 2012 [cited 2025 Aug 29];39:677–81. https://doi.org/10.1111/J.1346-8138.2012.01551.X

112. Uter W, Gefeller O, Geier J, Schnuch A. Methylchloroisothiazolinone/methylisothiazolinone contact sensitization: Diverging trends in subgroups of IVDK patients in a period of 19 years. Contact Dermatitis [Internet]. Blackwell Publishing Ltd; 2012 [cited 2025 Aug 29];67:125–9. https://doi.org/10.1111/J.1600-0536.2012.02075.X,

113. Schnuch A, Lessmann H, Geier J, Uter W. Contact allergy to preservatives. Analysis of IVDK data 1996–2009. British Journal of Dermatology [Internet]. Oxford Academic; 2011 [cited 2025 Aug 29];164:1316–25. https://doi.org/10.1111/J.1365-2133.2011.10253.X

114. Ackermann L, Aalto-Korte K, Alanko K, Hasan T, Jolanki R, Lammintausta K, et al. Contact sensitization to methylisothiazolinone in Finland—a multicentre study. Contact Dermatitis [Internet]. John Wiley & Sons, Ltd; 2011 [cited 2025 Aug 29];64:49–53. https://doi.org/10.1111/J.1600-0536.2010.01811.X

115. Toledo F, García-Bravo B, Fernández-Redondo V, De La Cuadra J, Giménez-Arnau AM, Borrego L, et al. Patch testing in children with hand eczema. A 5-year multicentre study in Spain. Contact Dermatitis [Internet]. John Wiley & Sons, Ltd; 2011 [cited 2025 Aug 29];65:213–9. https://doi.org/10.1111/J.1600-0536.2011.01943.X
